# Nanomaterials in Agriculture and Their Potential Hazards: A Review

**DOI:** 10.3390/nano16201281

**Published:** 2026-10-09

**Authors:** Patrycja Kabiesz, Dorota Palka, Anna Horzela-Miś, Jakub Semrau, Vinod V. T. Padil, Farshid Ghanbari, Stanisław Wacławek, Miroslav Černík, Mohammad Gheibi, Daniele Silvestri

**Affiliations:** 1 Faculty of Organization and Management, Silesian University of Technology, Roosevelta 26-28, 41-800 Zabrze, Poland; 2 JSW Szkolenie i Górnictwo Spółka z o.o., 44-336 Jastrzębie-Zdrój, Poland; 3 School of Nano Sciences (SNS), Central University of Gujarat (CUG), Kundhela, Vadodara 391107, India; 4 Research Center for Environmental Contaminants (RCEC), Abadan University of Medical Sciences, Abadan 6313833177, Iran; 5 Institute for Nanomaterials, Advanced Technologies and Innovation, Technical University of Liberec, Studentská 1402/2, 46117 Liberec, Czech Republic

**Keywords:** nanomaterials, sustainable agriculture, threats, environmental protection, risk factors

## Abstract

Nanomaterials are increasingly applied in modern agriculture to improve crop productivity, nutrient efficiency, and plant protection through fertilizers, pesticides, and delivery systems. However, their growing implementation raises significant concerns regarding environmental fate, ecological toxicity, and potential risks to human health. This review aims to (i) summarize current knowledge on nanomaterial applications in agriculture and (ii) critically evaluate their associated environmental and health risks. The manuscript integrates evidence on nanoparticle behavior in soil–plant–water systems, including transport, transformation, uptake, and accumulation across trophic levels. Particular attention is given to key toxicity mechanisms such as oxidative stress, membrane damage, genotoxicity, and bioaccumulation across terrestrial and aquatic ecosystems. The review also highlights limitations of current studies, which are predominantly short-term and laboratory-based, lacking long-term field validation and standardized risk assessment approaches. Emerging approaches such as artificial intelligence-based optimization and commercial nano-enabled agricultural products, along with existing regulatory frameworks, are further discussed. By integrating agronomic, toxicological, and regulatory perspectives, this review provides a comprehensive systems-level overview of nano-enabled agriculture, emphasizing the urgent need for harmonized risk assessment methodologies, long-term environmental monitoring, and interdisciplinary research.

## 1. Introduction and History of Nanomaterials

The classic definition of nanoparticles (NPs) traditionally defines them as particles with diameters ranging from 1 to 100 nm, or as those in a size distribution where at least half exhibit nanometric dimensions. In contrast, the term “nanomaterials” is broader and includes nanoparticles, nanofibers, nanotubes, nanorods, nanogels, nanoemulsions, and other nanoscale structures characterized by at least one dimension within the nanoscale range. Throughout this review, the term “nanomaterials” is used as the general category referring to all nanoscale materials applied in agriculture, whereas “NPs” specifically refers to particulate nanoscale structures. This distinction is applied consistently throughout the manuscript to improve terminological clarity and scientific precision.

The European Commission definition of nanomaterials is adopted as the primary reference in this study. The classical size-based definition (1–100 nm) is used here for descriptive and historical purposes, while the current EU definition is considered more comprehensive, introducing additional regulatory criteria such as particle number distribution and particle shape.

The European Commission first adopted a formal definition of a nanomaterial on 18 October 2011 (Recommendation 2011/696/EU) [1], which was subsequently updated by the Commission Recommendation of 10 June 2022 on the definition of nanomaterial (2022/C 229/01) [2]. Under the current definition, a nanomaterial is a natural, incidental or manufactured material consisting of solid particles, present either on their own or as identifiable constituent particles in aggregates or agglomerates, where 50% or more of the particles in the number-based size distribution fulfil at least one of three conditions: an external dimension in the size range of 1 nm to 100 nm, an elongated (rod-, fibre-, or tube-like) shape with two dimensions below 1 nm and the third above 100 nm, or a plate-like shape with one dimension below 1 nm and the others above 100 nm [2]. Relative to the 2011 recommendation, which used a volume-specific surface area (VSSA) greater than 60 m^2^/cm^3^ as a proxy indicator for inclusion as a nanomaterial [1], the 2022 revision removed this inclusion-based proxy and instead introduced a VSSA exclusion criterion, whereby a material with a VSSA below 6 m^2^/cm^3^ is not considered a nanomaterial [2]. The 2022 revision also eliminated the earlier option to apply a lower 1–50% number-size-distribution threshold in place of the standard 50% threshold, leaving 50% as the sole criterion [1,2]. The 2022 definition additionally introduced explicit conditions for elongated- and plate-shaped particles, broadening the scope of the earlier size-based criterion alone [2].

Beyond these definitional and regulatory criteria, nanomaterial behavior in the environment is also strongly governed by physicochemical factors: understanding nanoparticle fate in aquatic environments requires attention not only to particle size, but also to aggregation state and surface characteristics, as these factors jointly govern nanomaterial transport, reactivity, and ecotoxicological impact [3].

Against this regulatory and environmental backdrop, nanotechnology itself continues to expand rapidly as a scientific discipline. In today’s rapidly advancing landscape of technology and innovation, nanotechnology stands out as one of the fastest-growing scientific disciplines. Its development involves many other fields, including biology, molecular engineering, physics, and chemistry. These innovative technologies offer revolutionary solutions in materials science by enabling the design of materials with unique properties stemming from their nanometer-scale structures and precise molecular-level control.

**Figure 1:**
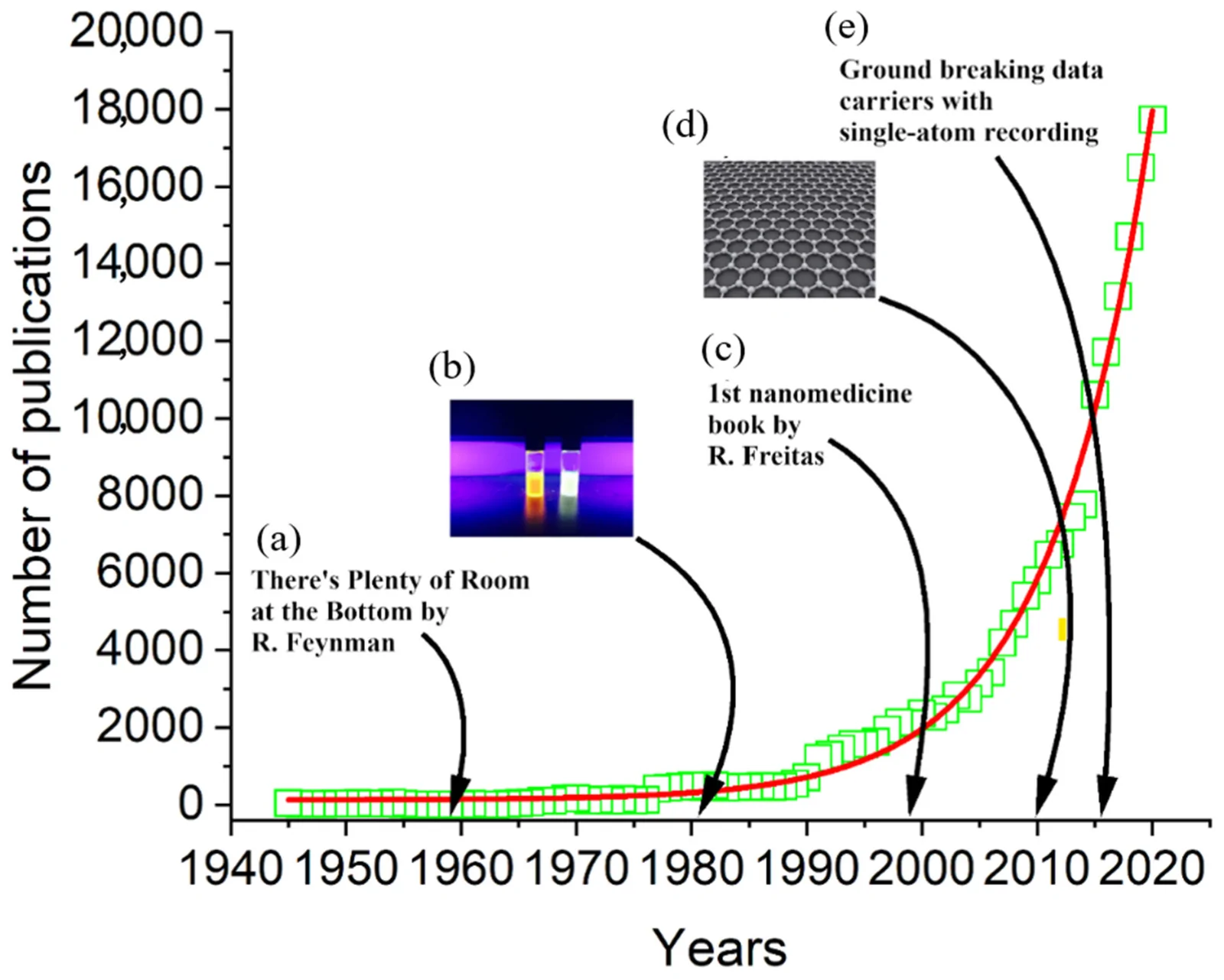
Number of published papers containing the keyword “nanotechnology” (WoS 2022). (a) R. Feynman presented the concept of miniaturization and the possibilities of using technology that can operate at the nanometer level, (b) Alexey Ekimov in 1981 was the first to synthesize Quantum Dots, (c) In 1999 R. Freitas published the first book on nanomedicine, (d) A. Geim and K. Novoselov got the Nobel Prize in Physics in 2010 and (e) in 2016 researchers at the Dutch Technical University in Delft have developed ground breaking data carriers with single-atom recording.

This growth traces its conceptual origins to Feynman’s visionary 1959 lecture, whose ideas have since become a reality in both laboratories and everyday life. Today, nanomaterials are widely used, and nanotechnology continues to unlock new possibilities for scientific research and practical applications (Figure 1).

**Figure 2:**
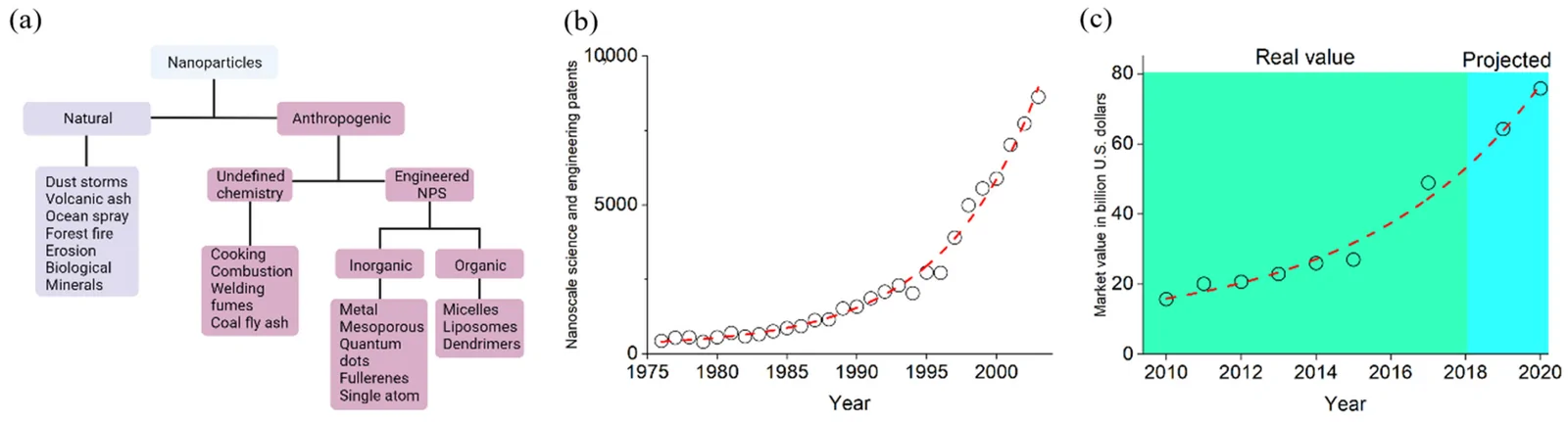
Classification and global development of nanotechnology: (**a**) classification of nanoparticles by origin, (**b**) international nanotechnology development in 2003 based on country, institution, and technology field analysis from the USPTO patent database, and (**c**) global market value of nanotechnology based on https://www.statista.com/statistics/1073886/global-market-value-nanotechnology/ (accessed on 15 April 2026).

While the conceptual history of nanotechnology traces the milestones shown above, the particles themselves long predate modern science. Particles in the nano-sized range have been present on Earth for millions of years, with only their anthropogenic (engineered) forms representing a comparatively recent addition [4]. Among natural sources of airborne particulate matter, wildfires are a well-documented contributor: forest fires introduce hazardous pollutants into the atmosphere and can substantially elevate ambient PM10 and PM2.5 concentrations above background levels during fire-affected periods [5]. However, many NPs emitted into the environment are formed as a result of human activity and are referred to as anthropogenic particles. Some are produced intentionally, while others are incidental byproducts associated with industrial growth and nanotechnology. NPs occur in locations where high-temperature processing takes place. Particles in the size range of several dozen nanometers are formed during cooking and frying processes. A common source of environmental NP pollution is combustion processes, such as emissions from heating plants, combined heat and power plants, household fuel combustion, and vehicle exhaust. In the case of exhaust emissions from diesel engines, where they result from incomplete combustion of diesel fuel, more than 90% of these particles have an aerodynamic diameter of less than 1 μm. The above-mentioned division of NPs is presented in Figure 2.

Industrial development, including the growth of the nanotechnology market, has led to an increase in the concentration of NPs in our environment [6]. Numerous reports indicate the negative impact of NPs on microbial, plant, and animal cells, highlighting the necessity for research on hazard assessment, exposure, and risk characterization. This need is especially pressing as NPs are increasingly used in the food and agricultural industries.

**Figure 3:**
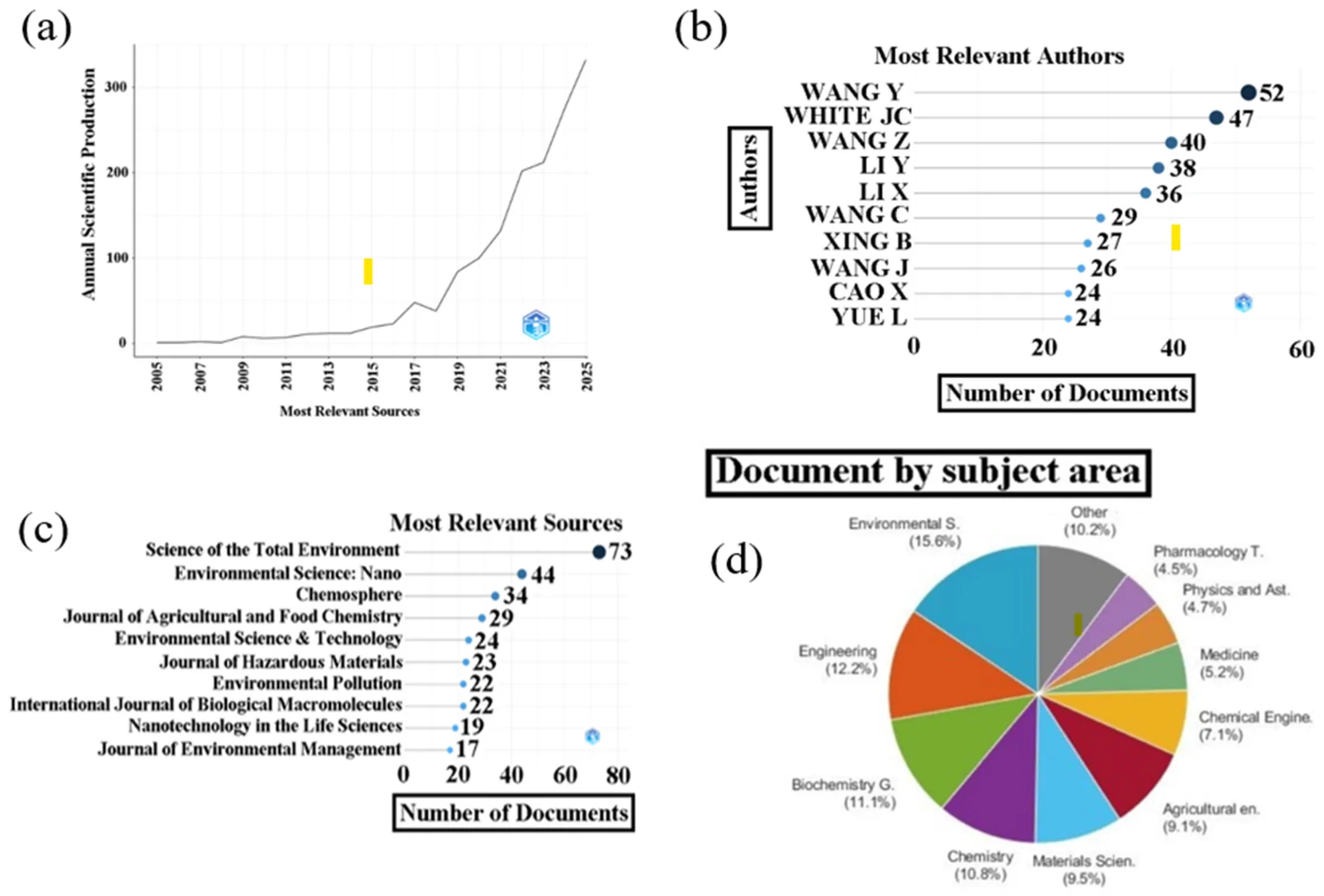

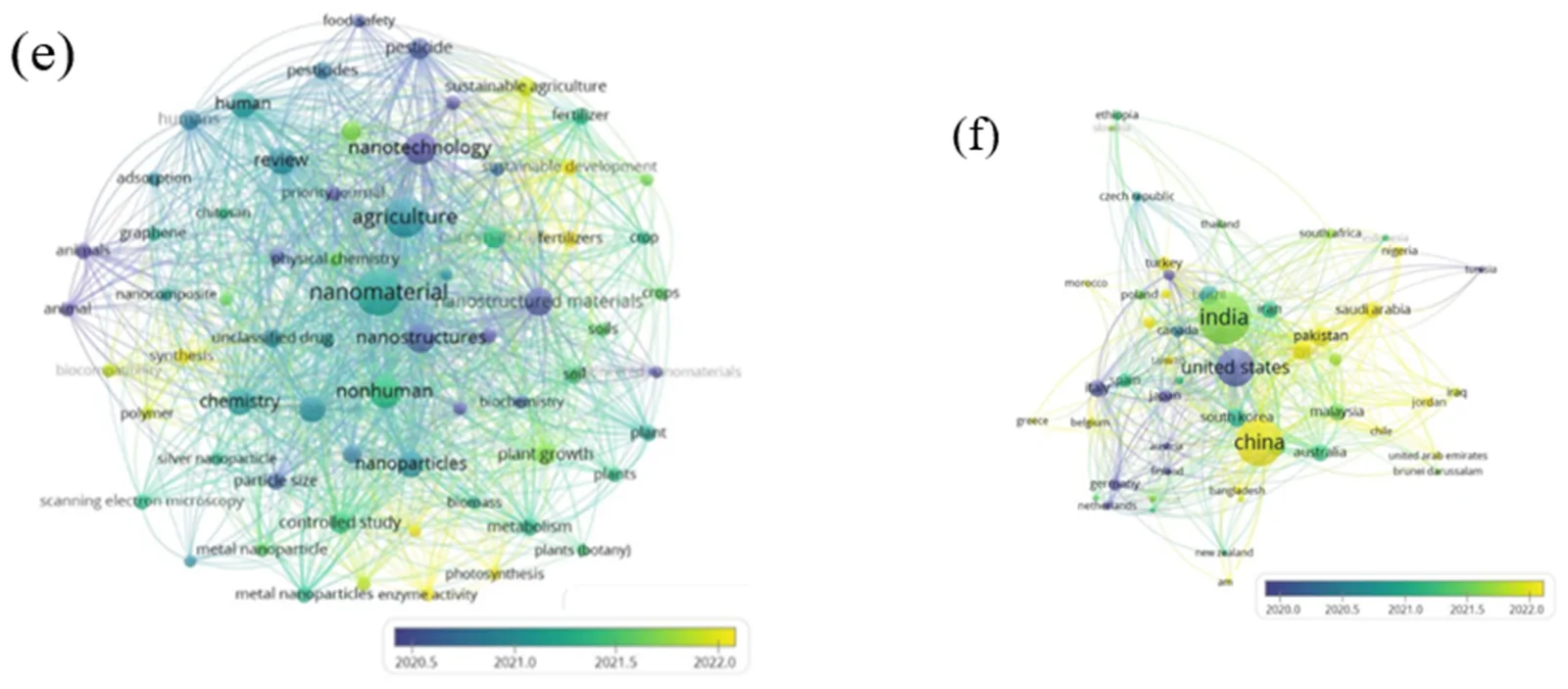
Bibliometric analysis of 1220 documents (2005–2025) with key words “Nanomaterials” and “Agriculture” from the Scopus database. (**a**) Document distribution by year (2005–2025), (**b**) top contributing authors, (**c**) document distribution by source, (**d**) subject area categorization, (**e**) keyword co-occurrence network in range with high repetition (VOSviewer), and (**f**) international research collaboration network (VOSviewer).

To quantify the scale and trajectory of this growing research interest, a bibliometric analysis of the literature was performed, as presented in Figure 3. Figure 3 presents a comprehensive bibliometric analysis of 1220 documents (“Nanomaterials” AND ”Agriculture”) published between 2005 and 2025, extracted from the Scopus database. The analysis is divided into six subfigures (a–f), where Figure 3a–d are derived directly from the Scopus data bank, while Figure 3e,f are generated using Scopus data mining with VOSviewer software (version 1.6.20). Additionally, the Biblioshiny application implemented in R was used to perform bibliometric analysis based on the Scopus dataset, enabling visualization and evaluation of research trends, authors, keywords, and scientific collaboration networks.

Figure 3a illustrates the trend in the number of documents published per year. The trend indicates a gradual increase in publications from 2005 to 2017, followed by a sharp rise from 2017 onwards. The number of documents reached approximately 210 by 2023 and continued to climb, surpassing 300 publications by 2025. This suggests a strong and sustained increase in research interest in the subject over the last decade, with no indication of a slowdown through 2025.

Figure 3b presents the top 10 contributing authors ranked by the number of documents. Wang Y. leads with the highest number of publications, followed by White JC., and Wang, Z. Other notable contributors include Li Y. and Li X., and several others. This highlights the most prolific researchers in the field and their significant contributions to the scientific literature.

Figure 3c compares the number of documents published across different sources. Key journals such as Environmental Science Nano, Environmental Pollution, Chemosphere, Environmental Science & Technology, and Science of the Total Environment are considered.

Figure 3d categorizes the documents based on subject area. The largest share belongs to Environmental Science (15.6%), followed by engineering (12.2%), biochemistry (11.1%), and chemistry (10.8%). Other subject areas, including materials science, agricultural science, chemical engineering, medicine, physics, and pharmacology, contribute to a smaller percentage of publications. This distribution highlights the multidisciplinary nature of the research field, with a strong focus on environmental and engineering aspects.

Figure 3e provides a co-occurrence network visualization of keywords extracted from the Scopus database using VOSviewer software. Larger nodes represent frequently occurring keywords, and connections between them indicate co-occurrence relationships. Key terms such as nanomaterials, nanotechnology, agriculture, chemicals, food safety, and environmental science appear prominently, suggesting the major themes explored in the literature. The color gradient reflects the timeline of keyword occurrences, indicating the evolution of research trends over 2020–2022.

Figure 3f visualizes country collaborations in research publications. Larger nodes represent countries with higher publication output, while connecting lines depict collaborative research links. India, the United States, and China appear as the most dominant contributors, with strong collaborations with countries like Pakistan, the United Kingdom, and Saudi Arabia. The timeline gradient suggests that international research collaborations have intensified in recent years, with an increasing number of countries participating in research within this domain. The prominent contribution of China, India and United States reflects the rapid expansion of nanotechnology research in countries with large-scale and highly intensive agricultural sectors.

Consistent with this bibliometric pattern, China has become an increasingly important contributor to research on agricultural nanotechnologies [7], including the development of nano-enabled pesticide delivery systems [8]. Experimental work has shown that flooded rice cultivation systems, through redox and pH shifts during flooding–drying cycles, can significantly influence the bioavailability of nanoparticle-associated metals in paddy soil [9]. Researchers have also emphasized persistent gaps in knowledge regarding the long-term ecosystem impact and regulatory oversight of nanomaterials in agriculture [10,11].

Beyond these specific gaps, current knowledge remains highly fragmented and often focused on isolated aspects, such as plant responses, nano-enabled fertilizers, or environmental toxicity alone. Long-term field-scale assessments and standardized risk evaluation approaches remain insufficiently developed. An integrated and interdisciplinary review is therefore needed.

The main objective of this review is to critically evaluate the current state of knowledge regarding the applications of nanomaterials in agriculture and their potential environmental and health hazards. Specifically, this study aims to: (i) summarize the major agricultural applications of nanomaterials, including nano-fertilizers, nano-pesticides, and nano-delivery systems; (ii) analyze the environmental behavior, transport, transformation, and accumulation of nanomaterials in soil–plant–water systems; (iii) evaluate the toxicological mechanisms associated with nanoparticle exposure in humans, animals, plants, and microorganisms; and (iv) discuss current regulatory frameworks, economic implications, and emerging technological perspectives, including artificial intelligence-assisted optimization of nano-enabled agricultural systems.

Building on these objectives, this review further contributes a bibliometric analysis of global research trends, providing a quantitative perspective that complements the mechanistic, economic, and regulatory synthesis outlined above. By integrating these aspects, this review highlights current challenges, identifies important knowledge gaps, and discusses future research directions supporting the safe, responsible, and sustainable implementation of nanomaterials in agriculture.

## 2. Methodology

This study was conducted as a structured literature review focusing on the application, environmental behavior, and risks of nanomaterials in agricultural systems. To improve transparency and reproducibility, a systematic search strategy was implemented, informed by PRISMA-inspired principles (Preferred Reporting Items for Systematic Reviews and Meta-Analyses).

### 2.1. Data Sources and Time Frame

The literature search was performed in the Scopus database, which was selected due to its wide coverage of peer-reviewed scientific journals in the fields of environmental science, nanotechnology, agriculture, and toxicology. The search was conducted between May and September 2025, focusing on publications from the past decade up to 2025 in order to capture both foundational and the most recent developments in nanotechnology applications in agriculture.

In addition to Scopus, supplementary searches were performed in Web of Science and Google Scholar to ensure inclusion of relevant studies not indexed in Scopus, particularly recent review articles and highly cited experimental studies.

### 2.2. Search Strategy

A combination of keyword groups was used to identify relevant literature. The search terms were applied using Boolean operators (AND/OR) and included:

- “nanoparticles” OR “nanomaterials” OR “nanotechnology” AND
- “agriculture” OR “agricultural systems” OR “crop production” AND
- “nano-fertilizers” OR “nano-pesticides” OR “nanoherbicides” OR “nanotoxicity” OR “environmental risk”

Additional targeted searches were performed using specific terms such as:

- “ROS AND nanoparticles”
- “soil nanoparticle transport”
- “food chain bioaccumulation nanoparticles”
- “ecological risk assessment nanomaterials”

### 2.3. Study Selection Criteria

The selection of studies followed a predefined two-stage screening process:

1. Inclusion criteria:
  - Peer-reviewed journal articles
  - Studies written in English
  - Publications focusing on nanomaterials in agriculture, environmental fate, toxicity, or risk assessment
  - Experimental, review, or modeling studies
2. Exclusion criteria:
  - Non-peer-reviewed sources (e.g., conference abstracts without full text)
  - Studies not related to agricultural or environmental applications
  - Duplicate records
  - Publications lacking sufficient methodological or experimental detail

### 2.4. Screening and Selection Process

The selection process was conducted in three steps:

1. Identification: Initial database search returned all records based on keyword combinations.
2. Screening: Titles and abstracts were reviewed to remove irrelevant studies.
3. Eligibility: Full-text articles were assessed for relevance and methodological quality.

The screening procedure was designed to ensure consistency and transparency in study selection. 

Disagreements during screening were resolved through consensus between the authors.

### 2.5. Data Extraction and Synthesis

From the selected studies, the following information was extracted:

- Type of nanomaterial
- Agricultural application (fertilizers, pesticides, soil amendment, etc.)
- Environmental behavior and fate
- Toxicity endpoints (plants, soil organisms, animals, humans)
- Risk assessment findings

The extracted data were qualitatively synthesized to identify common mechanisms, trends, and knowledge gaps in the field. Due to the heterogeneity of study designs and measured endpoints, a meta-analysis was not performed.

### 2.6. Methodological Limitations

Although a systematic approach was applied, some limitations should be acknowledged. The reliance on database indexing may have excluded certain relevant studies, particularly recent publications not yet indexed in Scopus at the time of analysis. Furthermore, variability in experimental conditions across studies limits direct comparability of quantitative results. In addition, because this review was not designed as a full PRISMA-compliant systematic review, no PRISMA flow diagram or quantitative summary of the study selection process is presented. Consequently, the findings should be interpreted as a structured qualitative synthesis of the available literature rather than as the results of a formal systematic review.

## 3. Nanomaterials in the Agricultural Industry

Nanotechnology plays a crucial role in sustainable agriculture [11] because it enables precise control over nutrient and pesticide delivery and facilitates soil quality monitoring and water resource management [12]. In addition, nanotechnology is an integral part of resource management in arable land, plant drug delivery systems, and soil fertility maintenance [13]. Nanomaterials are also increasingly applied in food processing, preservation, packaging systems, and smart sensing technologies used in the agri-food sector [14]. 

**Figure 4:**
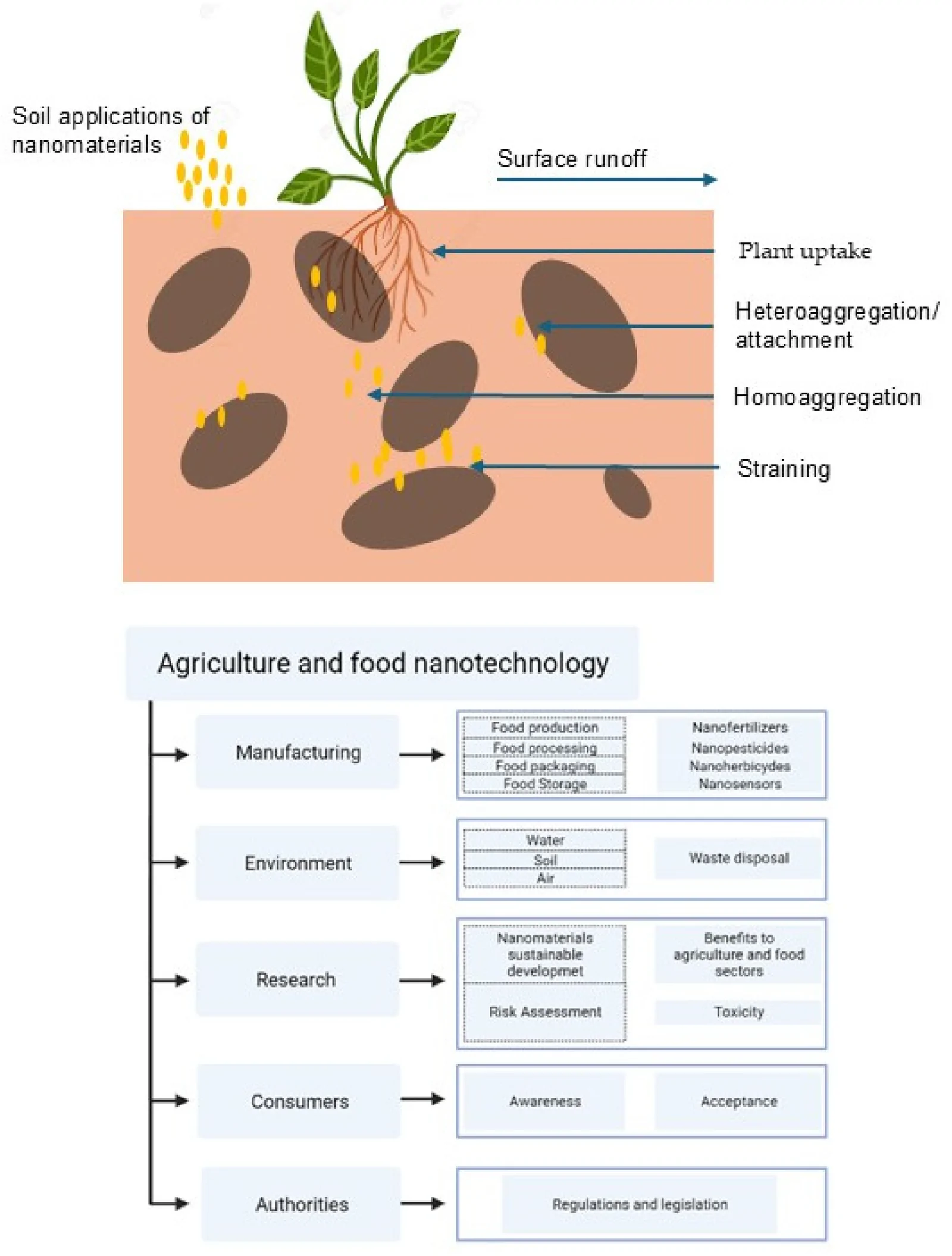
Fate of nanomaterials in soil system. The impact of nanomaterials on the environment in agriculture and food production. Based on [7,8].

The various nanomaterials introduced demonstrate great potential across all facets of the food industry, from agriculture to processing and consumption. Figure 4 illustrates not only the impact of nanomaterials in agriculture and food production but also their fate in the soil system, including their environmental transformation, mobility, bioavailability, and potential ecotoxicological effects.

The application of nanotechnology in agriculture and food production is relatively recent compared to medicine, pharmaceuticals, and electronics; hence, its environmental impacts remain insufficiently studied. Furthermore, the environmental behavior and toxicological effects of nanomaterials strongly depend on their physicochemical properties, including particle size, morphology, surface charge, aggregation state, and chemical composition.

Nanomaterials used in the agricultural industry can broadly be classified into metallic nanoparticles (e.g., Ag, Au, ZnO, Fe_2_O_3_), carbon-based nanomaterials, and polymeric nanomaterials, each offering distinct properties suited to specific agricultural applications [15]; carbon-based materials and clay minerals are likewise recognized more broadly as classic examples of natural and engineered nanomaterials [16]. In agriculture, silver NPs have been highlighted as a tool with the potential to support disease detection, pest control, and increased livestock production [17], and more broadly they are the most commonly used nanomaterial overall due to their broad-spectrum antimicrobial properties [18]. Furthermore, silver NPs are widely used for sterilizing medical devices due to their strong antimicrobial properties [19]. The coating and surface characteristics of nanoparticles play an important role in determining their stability and interactions within plant and agricultural systems [20].

However, only a few substances in nanoform have reached commercial food contact applications, especially in the EU, mainly because of legislative hurdles and uncertainty about their efficacy and economic and environmental impact, and demonstrating the absence of migration into food may facilitate their regulatory and consumer acceptance [21].

Most identified nanomaterial applications are in food (88%), followed by agriculture (9%) and feed (3%) [21]. Nanomaterials are increasingly being used in agriculture [22]. 

However, the unique properties of nanomaterials lead to diverse and complex interactions with biological systems that are difficult to monitor, and the complexity of environmental conditions makes it difficult to predict their behavior. A structured, multidisciplinary risk-assessment framework is therefore recommended, with targeted evaluation tests on specific organisms essential for assessing environmental nanotoxicity [23]. NPs can be released into the environment—including into soil, water, and air—both intentionally and unintentionally [24,25]. Due to their small size and high surface area to volume ratio, NPs usually show an extremely high reactivity potential.

According to Arruda et al. [20], various possible mechanisms explain the behavior of NPs in plants. For instance, NPs may dissolve directly in the soil, releasing ions that the plant system can absorb. Additionally, they can fragment in the soil into smaller NPs, which may then be taken up by the plant.

While numerous studies exist on the toxicity of other metals in plants, specific research on NP responses remains limited. Future investigations should emphasize the effects of NPs on plant–soil–microorganism interactions, prioritizing studies under realistic soil conditions and employing multidisciplinary approaches. Furthermore, exploring genetic variability, including the use of mutants and transgenic plants, is crucial, as different genotypes of the same species may exhibit markedly different responses to the same NPs [20].

In the following sections, selected nanomaterials, including CNTs, Quantum Dots (QDs), nanorods, core-shell nanoparticles, nanoemulsions, and nanogels, are discussed as representative examples of structurally and functionally diverse nanosystems that have been extensively investigated or demonstrate considerable potential for agricultural applications. This selection is intended to provide a comparative overview of major nanomaterial classes currently attracting scientific and industrial interest in sustainable agriculture rather than to present an exhaustive classification of all nanoscale materials.

To improve the systematic classification and comparison of nanomaterials used in agriculture, Table 1 summarizes the major types of nanomaterials, their structural characteristics, physicochemical properties, and selected agricultural applications.

### 3.1. Carbon Nanotubes

CNTs were originally discovered by Sumio Iijima in 1991. Using an arc-discharge evaporation technique similar to that used for fullerene synthesis, he observed needle-like carbon structures with diameters of a few to a few tens of nanometers and lengths of up to about 1 μm, examined by electron microscopy—these structures are now known as CNTs [26]. Depending on their chiral structure, CNTs can behave as either metallic or semiconducting materials [27], a property exploited in electrical circuits and nanodevices. They consist of two-dimensional sheets of graphene rolled into a tube with a hexagonal structure that can vary widely in size, shape and structure. CNTs can be classified into single-walled CNTs (SWCNTs) and multi-walled CNTs (MWCNTs); MWCNTs consist of multiple layers of graphene with inner diameters of 1–3 nm, while SWCNTs are composed of a single graphene layer and have diameters of 0.6–1.4 nm. The maximum tensile strength reported for a carbon nanotube was 63 GPa [28]. Owing to their high surface area and sorption capacity, CNT-based materials have also been explored for environmental remediation applications such as water treatment and the removal of oil pollutants [29]. More broadly, nanotechnology-based devices such as nanosensors are being explored for precision farming, crop monitoring, and nutrient management in agriculture [30]. Within this context, single-walled and multi-walled CNTs specifically are being investigated as a slow-release system for fertilizers and pesticides and as biosensors for agrochemical ingredients [31]. More generally, controlled-release nanodevices can reduce pesticide use, leaching, and environmental impacts while improving application efficiency [31]. CNTs can be used to purify water from pesticides, fertilizers, oils, etc. They are characterized by high adsorption of toxic organics such as dichlorobenzene from water. Pyrzynska demonstrated that CNTs can be used as sorbents for the analysis of pesticides [32].

In addition, research studies have shown the importance of CNTs in helping bone growth. CNTs are incorporated into synthetic and natural polymer composites to enhance their mechanical properties for bone tissue engineering applications [33]. On the other hand, other research interactions taking place in cells or tissues with CNTs may have an adverse effect, translating into a threat to human health [34].

Furthermore, CNTs have attracted considerable interest in the agricultural sector due to their potential applications in improving seed germination and plant growth [35], with additional studies reporting mixed, tissue- and species-dependent effects on root and leaf development [36,37], as well as their ability to penetrate plant cell walls and enable intracellular delivery of substances; however, for the sake of brevity, CNTs will not be further elaborated in the remaining sections of this article.

### 3.2. Quantum Dots

QDs were first developed in the early 1980s, and the Nobel Prize in Chemistry 2023 was awarded for the discovery and development of QDs. Rapid technological development has contributed to enormous progress in the methods of producing and applying QDs in many fields [38].

Quantum dots are often synthesized via colloidal methods, involving controlled precipitation and crystallization from molecular precursors in solution. During synthesis, ligands are required to prevent QD aggregation and regulate their growth. Controlling particle size and obtaining a narrow size distribution are particularly important because QDs exhibit size-dependent electronic and optical properties [39].

QDs are semiconductor nanomaterials consisting of single crystals a few nanometers in diameter. They possess composition- and size-dependent absorption and emission properties [40]. QDs also act as photocatalysts for the light-driven chemical conversion of water into hydrogen as a route to solar fuel [41].

Due to their unique optical and electronic properties, QDs have been widely investigated as next-generation fluorescent markers, including conjugation to monoclonal antibodies and use in bioimaging systems [42]. Another promising application is to develop appropriate NP-based drug carriers that enable drug delivery to pathological cells [43].

QDs can be used for live imaging in plant to validate known physiological processes, since at low concentrations, they do not show any detectable cytotoxicity to seed germination and plant growth [44]. Recently, QDs have been gaining significant interest in agriculture to improve plant growth and production. Li et al. [45] reviewed the interactions of Carbon Dots (CDs) with plants, including their effects on plant growth, internal physiological processes, and other external factors affecting plant growth.

Fluorescent carbon Quantum Dots (CQDs) have unique and tunable properties. First discovered in 2004, CQDs are clusters of carbon atoms with diameters generally below 10 nm, and they contain significant proportions of oxygen and nitrogen. CQDs can exhibit strong luminescence and, in some cases, do not require complicated purification or passivation procedures; their emission properties can be fine-tuned by changing the synthesis conditions, which can affect their size and surface chemistry [46]. This offers a potential advantage over conventional semiconductor quantum dots, whose use as contrast agents in optical and diagnostic imaging has been limited by concerns over their stability and inherent cytotoxicity arising from the leaching of semiconductor metals into the biological environment [47].

Studies have shown that slight variations in reaction parameters during synthesis can lead to adaptations in the properties of the obtained CQDs. Moreover, carboxyl groups on CQDs can contribute to red-shifted emission, thereby extending the range of accessible emission wavelengths [48].

### 3.3. Nanorods

Nanorods are examples of 1D nanostructures with a width in the range of a few nanometers and a much longer length. Display technology is one of the applications of nanorods because the reflectivity of the rods varies depending on their orientation in a magnetic field. The physicochemical properties of gold nanorods are used to detect heavy metals, e.g., lead in agricultural soil [49], or for the production of high-performance substrates for pesticide detection using surface-enhanced Raman spectroscopy (SERS) [50], where gold nanorods and graphene were the key materials. Gold nanorods demonstrated the ability to transport the synthetic auxin 2,4-D, resulting in a significant effect on the regulation of tobacco cell culture growth [51].

### 3.4. Core-Shell NPs

Core-shell-NPs (CSNs) have attracted significant attention because of their unique features, including excellent catalytic, optical, and electronic properties. They are widely used in sensing, imaging, and medical applications and solve several issues related to agriculture [52,53].

CSNs are made by encapsulation of an active material or mixture by a coating material or system [54]. The encapsulation technique is well developed and widely used in the pharmaceutical, chemical, cosmetic, food, printing, medical and agricultural industries [55,56]. The benefits of encapsulation methods include protection of substances/objects from unfavorable environments, as well as controlled release and precise targeting [57].

Microcapsules can achieve controlled, gradual release that improves fungicide efficacy and persistence, providing better disease control than current commercial formulations [58]. Moreover, nanoencapsulation, a nanotechnology approach, can be used to improve insecticidal efficacy. Controlled release of the active ingredient can improve effectiveness while reducing pesticide input and associated environmental hazards [59].

### 3.5. Nanoemulsions

The production of nanoemulsions can require substantial energy input; high-energy methods rely on special devices capable of generating extreme shear stresses, such as high-pressure homogenizers or ultrasonic generators, while lower-energy methods (e.g., phase inversion techniques) are also used [60]. Due to the small diameter of the droplets, typically on the order of 100 nm, nanoemulsions scatter light weakly and are often perceived as transparent. Nanoemulsions allow functional ingredients to be introduced within the dispersed phase droplets, adsorbed at the interfacial layer, or dissolved in the continuous phase. Consequently, they are used in delivery systems for active substances such as β-cypermethrin in pesticides [61]; comparable nanocarrier platforms (cationic nanoemulsions, liposomes, dendrimers) are also used to deliver mRNA vaccines [62]. More broadly, nanotechnology-based delivery systems enable the introduction of effective pesticides, plant growth regulators, and chemical fertilizers to crops [63,64,65]. Such delivery can be achieved by formulating active ingredients at the nanoscale and incorporating them into solid lipid carriers or other nanoscale delivery formats [66]. In addition, nanoemulsions serve as delivery tools for genetic material in plants, contributing to the development of an increasing number of genetically modified plants. Overall, nanoemulsion-based platforms in agricultural production offer a versatile route for delivering pesticides, fertilizers, and genetic material with improved stability and targeted release.

### 3.6. Nanogels

Due to their unique properties, nanogels are of great interest, particularly in biomedicine and pharmaceutics, as evidenced by a large number of publications on their preparation, properties, and applications [67,68,69,70].

Nanogels are typically in granule form. They have a three-dimensional structure formed by chemically or physically crosslinked polymers with hydrophilic or amphiphilic macromolecular chains. These structures are able to expand and hold a high amount of water. Their most important feature is maintaining structural integrity without dissolving. The high water content is associated with fluid-like transport properties for active molecules that are much smaller than the gel pore size [71].

The presence of nanogels in the soil has a positive effect on nutrient uptake by roots, water transport, and thus on plant development [72]. Pheromone nanogels containing methyl eugenol are used to control pests on plants [73], while nanogels loaded with ash NPs or inorganic metal NPs have been shown to exhibit insecticidal, antimicrobial, and antifungal properties. In addition to protecting crops and food products from pests, nanogels are used to increase crop yield and quality [74] by improving nutrient use efficiency, thereby allowing a reduction in the amount of fertilizers applied.

## 4. Functions of Nanomaterials in Agriculture

Nanomaterials enhance agriculture by improving nutrient delivery, pest control, and plant growth. They enable targeted fertilizer release, increase pesticide efficiency, and boost soil quality. Additionally, nanomaterials aid in disease detection and stress resistance [20]. However, despite their considerable agricultural potential, the increasing use of nanomaterials also raises concerns regarding environmental accumulation, ecotoxicity, food safety, and long-term impacts on soil ecosystems. Therefore, both the beneficial and potentially hazardous effects of nanomaterials should be considered simultaneously when evaluating their agricultural applications. In the following, nano-pesticides for pest treatment, nanoherbicides, nanofungicides, and nano-fertilizers are evaluated.

### 4.1. Nano-Pesticides for Pest Treatment

In 2012, Khot et al. [22] described the use of nanomaterials in agriculture, particularly for plant protection and production, as an under-explored research area, noting that nanopesticides can improve the dispersion and wettability of agricultural formulations, reduce unwanted pesticide movement, and offer a large specific surface area with increased affinity to the target. In 2016, a nanocapsule pesticide formulation was developed with the properties of a slow release of active substances, increasing pest control effectiveness over a more extended period [75]. This pesticide product reduced chemical doses and human exposure, making it environmentally friendly and protective of crops [75]. The development of non-toxic plant protection products and effective implementation systems increases global food production while reducing negative impact on the ecosystem [76]. In 2018, a study synthesized magnesium oxide NPs using *Chamaemelum nobile* flower extract and examined the effect of different concentrations on green peach aphids under greenhouse conditions [77]. Similar studies synthesized nanomaterials of copper oxide, zinc oxide, magnesium hydroxide and magnesium oxide using aqueous extracts of *Punica granatum* peel, *Olea europaea* leaves and *Chamaemelum nobile* flowers [78]. Screening studies have demonstrated the effectiveness of NPs in pesticides by increasing the mortality rate of aphids.

Resistance often results from point mutations that alter the structure of target proteins such as ion channels or receptors, changing their sensitivity to the active substance. Resistance can also arise through gene amplification, which increases the production of enzymes responsible for detoxifying the active substance [79]. Because pests reproduce rapidly, resistant individuals can spread quickly through a population, leading to an uncontrolled increase in resistant insects. To counter this, farmers conventionally rotate different chemical classes to delay resistance development, a strategy that NP-based formulations may help reduce the need for, owing to their distinct and often multi-target modes of action.

Despite these advantages, the widespread application of nano-pesticides may also pose environmental risks associated with nanoparticle persistence, bioaccumulation, toxicity toward non-target organisms, and disturbances in soil microbial communities.

Table 2 shows the use of selected NPs for pesticide production.

**Figure 5:**
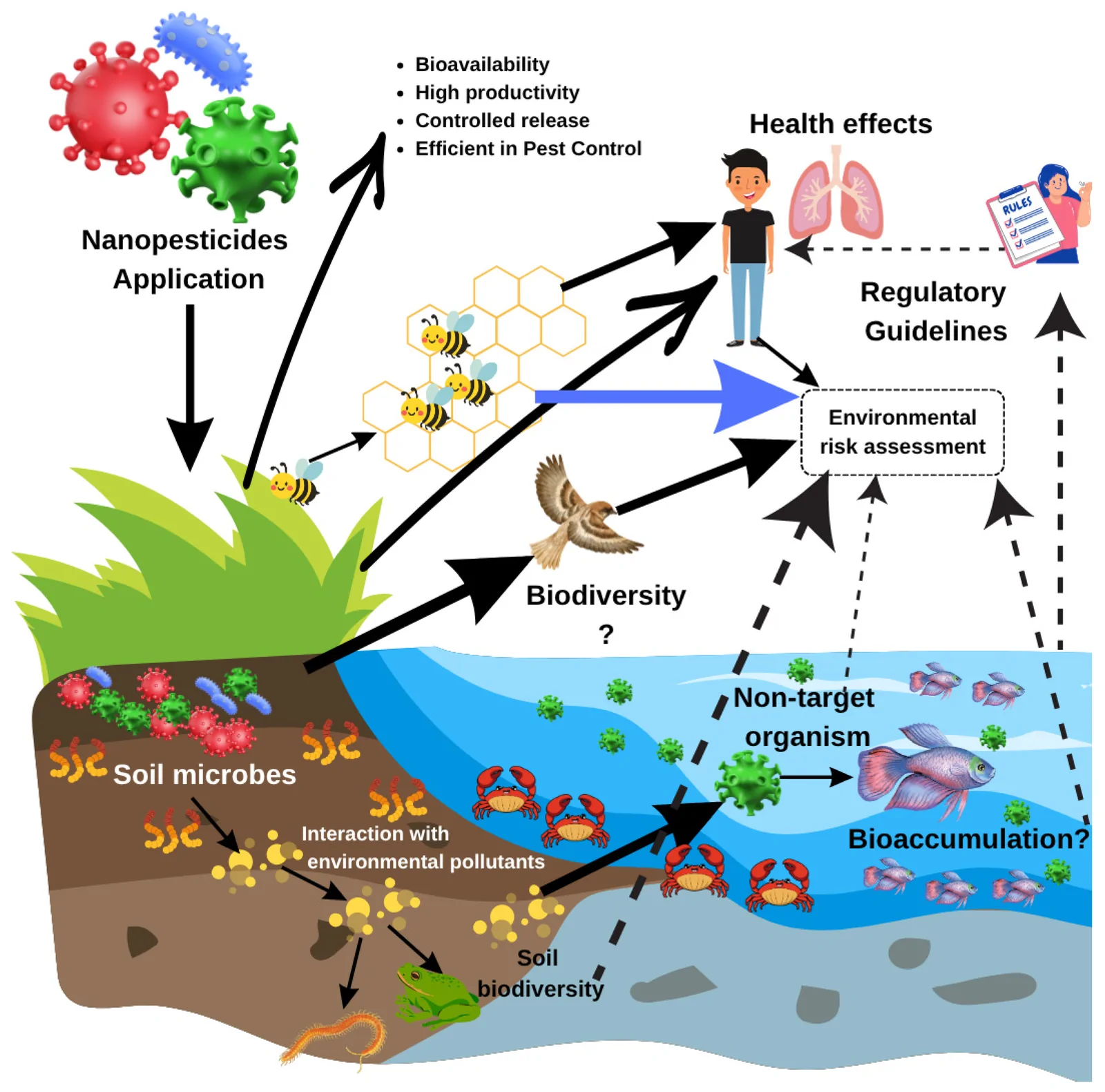
Environmental risk assessment of nano-pesticides in agriculture, adopted from: [96].

Figure 5 illustrates the benefits and potential risks associated with nanopesticide applications. These advanced pesticides offer enhanced bioavailability, controlled release, and high productivity, making them efficient in pest control. However, their interaction with soil microbes and environmental pollutants raises concerns regarding soil biodiversity and ecological balance. The potential bioaccumulation of nano-pesticides in aquatic ecosystems may affect non-target organisms, leading to biodiversity disruptions. Additionally, human exposure poses potential health risks, necessitating comprehensive environmental risk assessments and regulatory guidelines to ensure safe implementation in agriculture.

### 4.2. Nanoherbicides

The main purpose of herbicides application is to control unwanted plants and seeds in the soil and prevent them from germinating. Encapsulating herbicides in polymer core coatings of NPs improves the safety and management of chemicals with a lower environmental impact. It was noticed that during the fight against weeds, the phytotoxicity of herbicides on crops was reduced by using nanoencapsulated herbicides. Nanoparticle-based herbicide carriers have been proposed as a strategy to improve weed control efficacy and delay the emergence of herbicide-resistant weed populations [97]. Atrazine-containing nanocapsules are effective against the target *Brassica juncea* [98]. Nanoherbicides can mix with the soil, eliminate weeds in an environmentally friendly manner without leaving toxic residues, and prevent the growth of weed species that have become resistant to traditional herbicides.

For example, chitosan/tripolyphosphate NPs loaded with paraquat herbicide have been shown to enhance weed control efficiency while reducing the associated ecological footprint. Understanding the mechanistic interactions of such nanoherbicides with target organisms is crucial for optimizing their performance and minimizing unintended ecological consequences. Nanobased smart herbicide formulations offer emerging opportunities in agriculture, enabling controlled release, improved targeting, and reduced environmental contamination, thereby contributing to sustainable and precision farming practices [99,100,101].

### 4.3. Nanofungicides

Plant pathogens can be controlled to some extent by using resistant plant varieties and chemical treatments. Unfortunately, the emergence and development of new pathogens is a persistent problem, and the use of chemicals is expensive and not always effective. Fungal diseases pose a severe threat to the major crops on which humanity depends. If a major epidemic were to occur that severely reduced crop yields, its effects would spread throughout the global food system [102,103]. Recently, nanomaterials have started to be used as an alternative to pathogen control. The use of copper NPs acts against the phytopathogen *Colletotrichum gloeosporioides*. It has been found in research that metals (e.g., Ag and Cu) and metal oxides (e.g., CuO and ZnO) NPs are effective fungicides [104]. A well-dispersed and stabilized Ag NPs solution can serve as an excellent fungicide due to its strong adhesion to bacterial and fungal cell surfaces [105]. In addition, Cu NPs exhibit antifungal activity, with a mode of action that appears to differ from that of bulk copper compounds. They have also shown promising potential as nanofungicides, being more fungitoxic than a commercial copper hydroxide fungicide against most of the tested plant pathogenic fungi and, at the higher dose, suppressing grey mould symptoms on plum fruit more effectively than the reference fungicide [104]. However, despite promising antimicrobial activity, further investigations are required to evaluate their long-term environmental persistence, phytotoxicity, and potential effects on beneficial soil microorganisms.

### 4.4. Nano-Fertilizers

Fertilizers play a crucial role in increasing agricultural production, with long-term field trials estimating that commercial fertilizer use contributes to a 30–50% increase in crop yield [106]. In recent years, nano-fertilizers have been widely available on the market and are distinguished by their controlled release into the soil. NPs in fertilizers can improve crop yields and reduce environmental impacts compared with conventional fertilizers [107]. The influence of NPs on plants was investigated, including aluminum oxide, zinc, and zinc oxide affecting seed germination and root growth of the following plant species: radish, rape, ryegrass, lettuce, corn, and cucumber [108]. Silver NPs stimulate the growth and yield of wheat seedlings [109]. Zinc, often applied in the form of zinc oxide NPs (ZnO NPs), is a micronutrient essential for proper plant metabolic activity, even though it is used in trace amounts. It plays an important role in managing reactive oxygen species and protects plant cells against oxidative stress. Zinc deficiency reduces both yield and quality. Fertilizers containing chitosan NPs control nitrogen, phosphorus and potassium (NPK) release [110,111]. Foliar application of nanopotassium on *Ocimum basilicum* (basil) had a beneficial effect on its production [112]. The root elongation of *Lemna minor* was increased by Al_2_O_3_ NPs [113]. In addition, plant production increased significantly after the use of nanogels [72], and CNTs have shown mixed effects on seed germination and plant growth—inhibitory in some fodder grasses but beneficial in barley, soybean, and corn [114,115].

Although nano-fertilizers may significantly improve nutrient use efficiency and reduce nutrient losses into the environment, concerns remain regarding nanoparticle accumulation in soil systems, possible phytotoxicity at high concentrations, and long-term ecological consequences. Therefore, future studies should prioritize field-scale investigations, standardized safety assessments, and evaluation of nanomaterial interactions within complex soil–plant–microorganism systems.

## 5. Agricultural Release of the NPs into the Environment

In modern agriculture, achieving sustainable production and efficiency is possible due to the use of nanotechnology in pesticides, herbicides, fertilizers, and other applications [116,117]. However, nanoparticle-based agrochemicals also carry risks, including the potential translocation of NPs into edible plant parts, which may endanger human health [118]. Proper management and control of inputs can reduce the risk of adverse effects [65].

**Figure 6:**
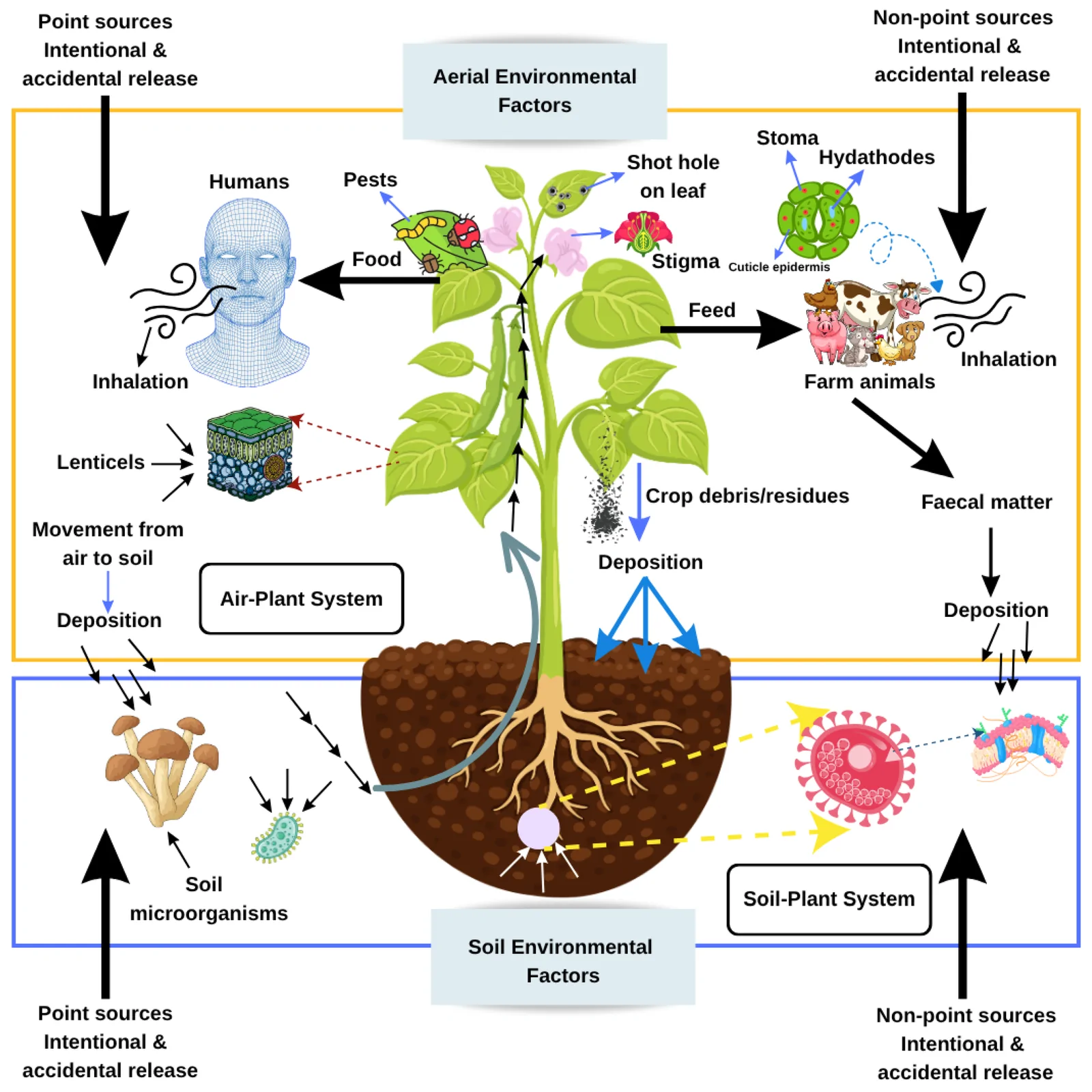
Agricultural sources of nanomaterials in the environment. Based on: [121].

Moreover, the rapid development and widespread use of NPs in agriculture carries the risk of the uncontrolled release of these compounds into the environment. This could have a significant impact on ecosystems in air, soil, and water [119]. Typical sources of nanomaterials in the environment are presented in Figure 6. However, the full extent of this phenomenon remains poorly understood, mainly due to the still limited amount of information available. To better understand the scale of this issue, it is necessary to examine more closely the toxicity of nanomaterials in air, soil, and water, and how they affect ecosystems and aquatic life [120].

Nanomaterials released into the environment often act as contaminants. In both soil and aquatic ecosystems, microorganisms are typically the first living organisms to come into contact with these substances. Although data on the impact of nanomaterials on microbial communities remain limited, growing scientific interest highlights the need for further research in this area.

Holden et al. [122] give five reasons why it is essential to consider microorganisms in this context, namely:

- The potential reduction in microbial biodiversity,
- Alterations in bacterial physiology, which can affect nutrient cycling,
- Microorganisms’ ability to modify the physical properties of nanomaterials,
- The potential for microbial degradation of nanomaterials,
- The role of microbes in transferring these compounds to higher trophic levels.

Handy et al. [123] highlight the need to refine current research approaches to better understand how nanomaterials interact with living organisms under real-world conditions. Key limitations include the omission of mechanical interactions and the difficulty of extrapolating laboratory results to complex environmental systems.

In the context of soil research, the authors stress the importance of considering nanomaterial absorption into soil particles and suggest evaluating material availability across the largest possible surface area. In aquatic environments, a significant obstacle is the tendency of certain nanostructures to aggregate and precipitate, which hampers the reproducibility of results [123]. Moreover, nanomaterials may behave differently in natural ecosystems compared to controlled laboratory conditions.

Given that agricultural soils are major receiving environments for NPs introduced through agricultural activities, the following section provides a brief overview of the behavior, transport, and fate of NPs, as summarized in the recent review by Chen [118].

Metal NPs enter agricultural systems through both direct and indirect pathways. Among indirect sources, sewage sludge applied to land is considered a significant contributor [118]. Sewage sludge retains and accumulates NPs; later, as sewage sludge is often used as fertilizer and soil conditioner, these NPs are released into the soil. Direct input, on the other hand, comes from nano-enabled agricultural products such as nano-fertilizers and nano-pesticides.

Once in the soil, metal NPs often tend to agglomerate into larger aggregates, losing their nanoscale properties and, consequently, much of their reactivity [124]. These aggregated NPs can become immobilized in the soil matrix. However, NPs that do not readily form large aggregates may remain mobile and travel long distances, potentially posing greater environmental risks. Thus, aggregation is a key factor influencing the mobility, reactivity, and environmental impact of NPs.

Dissolved organic matter (DOM), which consists of various organic compounds such as hydrophilic acids, amino acids, humic acids, and fulvic acids [125], plays a significant role in NPs behavior. The primary sources of DOM in agricultural soils include humus, root exudates, plants, and applied organic amendments. DOM can influence NPs aggregation in both directions—either enhancing or inhibiting it. For instance, humic acids have been shown to reduce aggregation in iron oxide (FexOy) NPs, as reported by Dickson et al. and Chekli et al. [126,127]. Similarly, Ghosh et al. [128] observed the effects of humic acids on aluminum oxide (Al_2_O_3_) NPs. On the other hand, studies by Mohd Omar et al. [129] and Baalousha et al. [130] reported the influence of DOM in NPs aggregation.

Understanding the transport of NPs in soil is essential to assess their potential impact on agricultural systems. The NPs transport in soil is governed by the filtration rate through the porous media; as particle size increases, their mobility decreases. Mobility is also influenced by size and porosity of the soil. Overall, the mobility of NPs decreases when the soil median grain size decreases. Other important parameters influencing NPs mobility are temperature, ionic strength, and pH.

The behavior of NPs in soil is highly variable and strongly dependent on their chemical composition. After adsorption to soil particles, some NPs become inert, while others retain or enhance their toxic effects. For example, fullerene C60 can undergo enzymatic biodegradation, showing that its persistence is not absolute [131]. Some engineered NPs may undergo biodegradation and experience changes in their chemical or physical characteristics, although the exact mechanisms behind these processes remain largely unknown. Most NPs used in nanotechnology are produced from poorly degradable materials, including ceramics, metals, metal oxides. Notably, C60 fullerol has been shown to be metabolized by white-rot fungal species [132], indicating that biodegradation pathways may exist under specific biological conditions. Some NPs undergo chemical transformations in the environment, e.g., nano-Fe^0^, which oxidizes to iron oxides. The largest threat may be free, single NPs due to their ability to penetrate most biological barriers (e.g., blood–brain barriers) [133].

Klitzke et al. [134] showed that bare Ag NPs sorb organic matter, with short-chained organic matter being preferentially adsorbed over long-chained, aromatic organic matter. Stabilizing effects of organic matter only come into play at higher Ag NPs concentrations [134]. Previous research has also shown that plants can absorb and translocate foliar-applied nanomaterials, affecting physiological stress responses [135]. Ge et al. [136] found a negative effect of TiO_2_ and ZnO NPs on the biodiversity of the soil microorganisms community.

**Figure 7:**
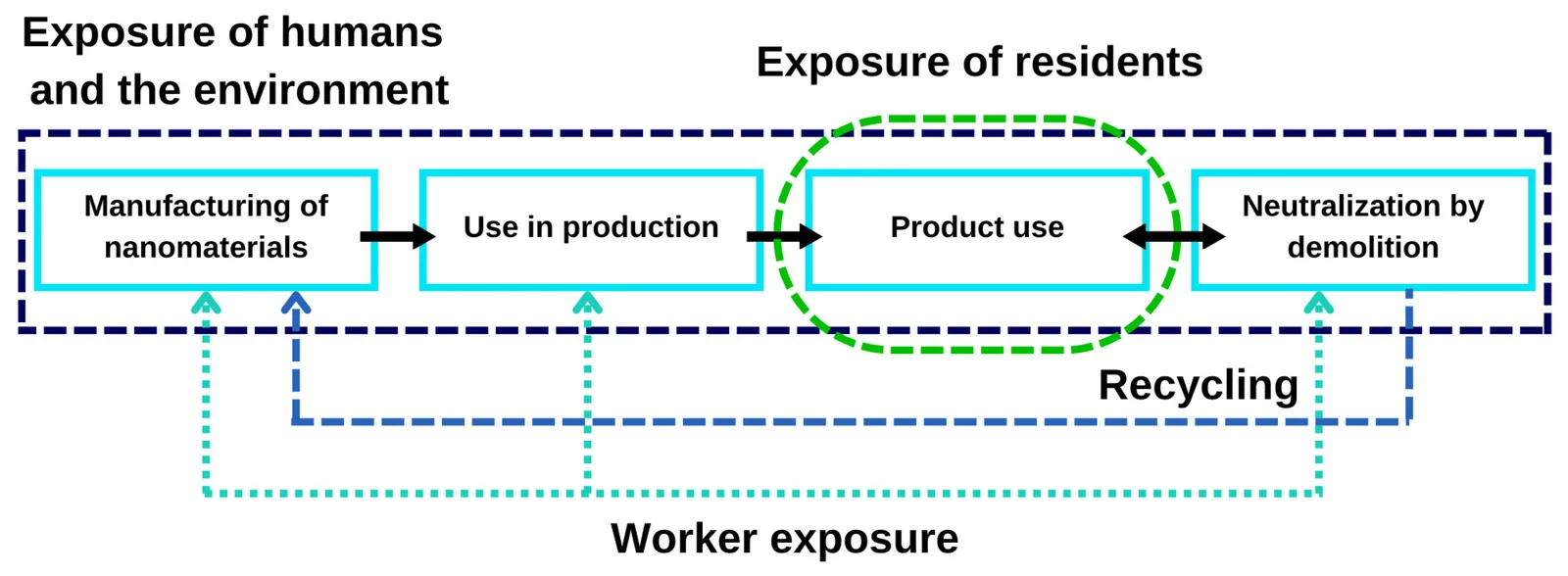
The release of nanomaterials into the environment. Based on: [137].

The use of nanoproducts is directly linked to creating a new category of waste, called nanowaste. This term refers to waste that contains materials with dimensions that characterize the nanoscale. Nanomaterials can be released into the environment at any stage of the product life cycle, from manufacturing processes, use for disposal or recycling (Figure 7) [137].

From this perspective, nanowaste management is a new challenge that is currently attracting the attention of many scientists. This issue underlines the need for continuous monitoring of the fate of nanoproducts. Notably, waste recycling processes (e.g., shredding of waste electrical and electronic equipment) represent a potential nanowaste release pathway that remains largely unstudied and warrants further investigation [138]. In addition, research into the disposal of nanowaste is necessary to reduce the unintentional release of nanomaterials into the environment. However, current monitoring and disposal technologies suffer from limited accuracy and a lack of comprehensive data, which hampers precise assessment of nanomaterial emissions [139]. Furthermore, the current level of voluntary industry involvement in ensuring the safe use of nanomaterials is insufficient. Although many applications of nanomaterials may be safe, the possibility of exposing consumers to an undefined risk still exists [140].

### 5.1. Toxicological Aspects of NPs Used in Agriculture

The design, production, and application of nanomaterials are becoming increasingly prevalent across industrial technologies and everyday consumer products [141]. As nanotechnology rapidly advances, growing quantities of NPs are entering the natural environment [142]. Research in this area highlights the risks to human health and the environment caused by certain nanomaterials. One of the major challenges in assessing these risks is the limited understanding of NPs properties and their interactions with the environment. Current exposure risk assessment methods are generally considered inadequate and underscore the need for further scientific investigation [143]. A significant issue is the absence of comprehensive regulations regarding hygiene and safety standards during the production and use of NPs [139]. Another considerable research problem is the proper selection of measuring equipment enabling the study of nanoscale particles, both their number in a given air volume and the mass of particles and their surface.

To address these challenges, the European Union has introduced specific regulatory provisions for nanomaterials under its chemical legislation. In particular, revisions to REACH (Registration, Evaluation, Authorisation and Restriction of Chemicals), in force since January 2020, establish nano-specific requirements for registration and environmental safety assessment, requiring manufacturers, importers, and downstream users to provide detailed information on nanomaterials before they can be placed on the market [144].

Research conducted in Europe and the USA has identified several risks associated with the use of NPs such as ZnO, TiO_2_ and Ag in living organisms [145]. For example, many studies have shown the harmful effects of ZnO NPs on plants, such as decreased biomass, inhibition of root elongation, and cytotoxicity in root cells [12,146], reduction in the root tip and epidermis, cortical cells undergoing severe vacuolization, inhibition of chlorophyll biosynthesis and decreased photosynthetic productivity [147].

Moreover, NPs found in terrestrial and aquatic environments are consumed by living organisms. They introduce foreign elements with distinct physicochemical properties, which can disturb natural physiological mechanisms and negatively affect both developing and adult organisms. NPs toxicity often manifests as malformations, which can ultimately lead to death [145]. While the chemical composition of NPs has a negative effect, their small size also adversely affects biological activity. Due to their tiny dimensions, NPs can easily penetrate living organisms unnoticed by defense mechanisms. Consequently, NPs can penetrate organelles such as mitochondria, where they cause cell death by altering metabolism [145,148].

### 5.2. The Influence of NPs on Humans

Nanomaterials used as food additives, functional food ingredients, and agricultural products are a concern for human health. As detailed in Section 5.4, ROS generation is the primary toxicity mechanism of metal-based NPs [149], disrupting redox homeostasis and leading to lipid peroxidation, protein denaturation, and mitochondrial dysfunction. Overproduction of ROS may further cause autophagy [150,151], DNA damage [151], and neurological disorders associated with aging [152]. Additionally, metal ions released from nanomaterials may trigger allergic reactions [153] and interfere with enzymatic activity at the tissue level.

**Figure 8:**
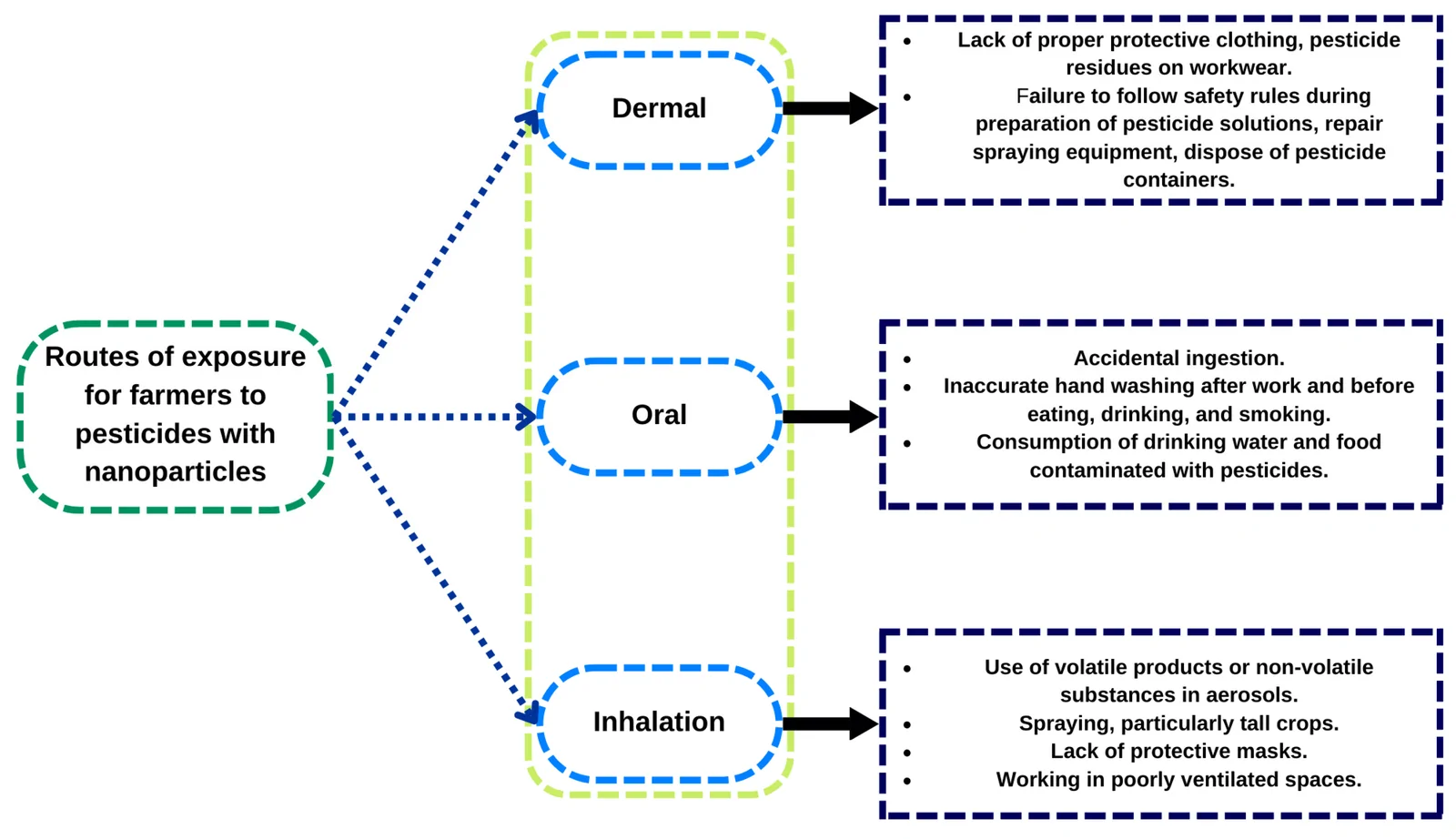
Routes of farmers’ exposure to nano-pesticides. Based on [154].

People who work in agriculture are particularly vulnerable to the negative effects of NPs used in many pesticides. Although such people are expected to follow all the rules of proper work with plant protection products to minimize the negative impact on human and animal health, the risk of exposure remains significant [154]. There are many situations in which harmful NPs can enter the body of a working farmer, as shown in Figure 8.

Currently used organophosphorus pesticides are highly fat-soluble, enabling them to easily penetrate the skin barrier. Dermal exposure is thus the most common route of exposure for agricultural workers to NPs-enriched agents. Pesticide carriers, i.e., NPs made of polymers, carbon-based materials, or inorganic NPs, are characterized by poor penetration through the skin barrier, passing through the epidermal layer only to a small extent and failing to reach deeper skin layers [155]. Active substances in pesticides are often released from their carriers and can penetrate the skin barrier. As a result of contact with the lipids contained in the skin, lipid NPs lose their stability, which may increase skin penetration by the active substance [156,157]. Any damage to the skin barrier increases the penetration of NPs into the bloodstream and their potential translocation throughout the body. Although NPs penetrate the skin poorly, their toxicity cannot be ruled out, as the effects of long-term exposure are unknown. Some NPs are antibacterial, so there is a risk that they will disturb the skin’s natural microflora. However, neither this phenomenon nor increased colonization of pathogenic microorganisms following skin exposure to NPs has been observed so far in human studies [158]. Once nanoparticles enter systemic circulation, they may distribute to secondary target organs such as the liver, kidneys, spleen, and brain, where they can induce local inflammation and oxidative stress.

Plant protection products can also enter agricultural workers’ bodies through inhalation. People who come into contact with pesticides have an increased risk of developing respiratory diseases, such as chronic bronchitis, bronchial asthma, and obstructive lung function impairment [159]. Contact with pesticides is also an environmental factor that stimulates the development of lung cancer [160]. Due to their low mass and small size, NPs stay in the air for a long time, and when inhaled, they can reach not only the upper but also the lower parts of the respiratory tract. At the alveolar level, nanoparticles can cross the air–blood barrier and enter systemic circulation, which significantly increases their toxicological potential. Metallic NPs are also dangerous. For example, nickel NPs induce a disruption of antioxidant processes in animal lung cells [161]. This disruption may lead to epithelial cell damage, chronic inflammation, and fibrotic remodeling of lung tissue, which are key pathological features of chronic respiratory diseases. In addition to being detrimental to the respiratory system, NPs can access the central nervous system. This system, in turn, is extremely susceptible to the action of harmful substances, leading to dysfunction or damage [162]. Neurotoxic effects may include impairment of neurotransmission, oxidative damage to neurons, and potential contribution to neurodegenerative disorders such as Alzheimer’s or Parkinson’s disease-like symptoms.

The last type of exposure is the oral route. Pesticides are absorbed along the entire gastrointestinal tract, with the most absorption occurring in the intestines. Animal studies indicate that orally administered silver NPs can enter the circulatory system and subsequently distribute to other organs. Oral exposure has also been associated with changes in liver enzyme activity. Following oral administration, silver was detected mainly in the liver and kidneys, followed by the testes and spleen [163]. In the liver, nanoparticle accumulation has been associated with hepatocellular injury, elevated transaminase levels, and impaired detoxification capacity.

### 5.3. Effect of NPs on Animals

It is now necessary to understand the direct or indirect threats resulting from the ubiquitous use of NPs. They are known to have an adverse effect on invertebrates, algae, mammals, and aquatic animals. Research is being conducted on living organisms to identify the impact of NPs on their functioning. Much research has shown adverse effects in mammals, including detrimental impacts on embryonic development and reproduction [164,165]. NPs can enter mammals through skin penetration and inhalation. Due to their small size NPs can move from one cell to another, potentially disrupting cellular functions. When inhaled, they can reach the olfactory nerve cells and subsequently the brain, where they may affect neurons; however, this process depends on several factors, including the size, surface properties, concentration of the NPs, and the physiological condition of the organism [166]. Another pathway into the body is through the lungs; from there, NPs may enter the bloodstream and travel throughout the body, although this process depends on various factors such as particle size, solubility, surface charge, and the condition of the respiratory epithelium. Research has shown that NPs can also reach the lymph nodes, heart, bone marrow, and spleen. These findings demonstrate that NPs, including metal oxides such as titanium dioxide (TiO_2_) and zinc oxide (ZnO), can cause oxidative stress and inflammation. Mammalian effects were dependent on the dose of NPs administered. Experiments carried out on mice and rats have shown a negative effect on brain function. Copper NPs with a diameter of 40 and 60 nm, administered at a low concentration of 1.5 µg/mL, cause proliferation of brain capillary cells, and at a higher concentration of 50 µg/mL cause an increase in prostaglandin E2 [167]. Other research has shown the adverse effects of NPs on the blood flow of the brains of mice and rats, which in turn caused cerebral edema [168].

As previously mentioned, silver NPs are increasingly used in agriculture, despite their adverse biological effects being known. Experimental studies show that the lungs and intestines can absorb silver NPs. Additionally, they can be absorbed through the skin [169]. Table 3 summarizes toxicological studies of NPs on animals, plants, and human cells across multiple agricultural and environmental exposure routes.

NPs can cause neurotoxic effects and reach individual organs, including the brain [185]. In inhalation studies, silver NPs mainly affect the liver and lungs, while in animals chronically exposed to silver NPs through food, effects were observed mainly on the liver [169]. A biologic monitoring study of silver-exposed workers estimated a time-weighted average occupational exposure of approximately 0.03 mg/m^3^ silver, based on silver levels measured in the blood, urine, and feces of 37 exposed workers compared with 35 unexposed controls; the study concluded that generalized argyria was unlikely to develop in workers exposed at this level [186].

The increasing accumulation of NPs in inland waters and seas has forced scientists to research aquatic organisms. These studies have led to NPs being considered a new category of water pollutants that can negatively affect aquatic organisms, similar to mammals, depending on their size. Therefore, research has focused on examining the effects of NPs on skeletal fish, sea urchins, crustaceans (e.g., *Daphnia* and *Thamnocephalus platyurus*), plankton, and mollusks [145]. At high concentrations of 100 μg/L and falling within the range of 3 and 220 nm Ag NPs, young salmon were exposed to gill plaque necrosis, as a result of which 73% of the specimens tested died [145]. The research focused on the mechanism of metabolism, excretion, distribution and absorption of substances in the fish’s organism compared to other chemicals. Fullerene C_60_ and TiO_2_ NPs were tested in the gastrointestinal tract, liver, adrenal glands, and gills. NPs penetrated tissues by transporting through cell membranes faster than metal ions or by diffusion. In addition, NPs in fish were rarely excreted by the kidneys, but they could be eliminated from the body along with bile. The effects of TiO_2_ and carbon NPs on trout hepatocytes were investigated, as well as those of fullerene C_60_, MWCNTs, and SWCNTs, both functionalized and non-functionalized. NPs with dimensions from 5 to 200 nm were used. Research indicates that some contaminants may show increased toxicity when combined with carbon nanoparticles, compared with exposure to the same contaminants in aqueous solution alone [187].

Crustaceans such as *Daphnia magna* and *Thamnocephalus platyurus* have been used to study the harmfulness of NPs in the environment. Chronic exposure of *D. magna* to silver nanoparticles primarily affected adult survival rather than reproduction. Notably, silver nanoparticles were up to ten times less toxic and less bioavailable to crustaceans than an equivalent concentration of dissolved silver (AgNO_3_) [188].

In addition, echinoderms and bivalve mollusks, which inhabit saltwater environments, are increasingly used to test NPs toxicity [145]. Studies on rock urchin have shown that SnO_2_, CeO_2_ and Fe_3_O_4_ NPs in immune cells cause a reduction in esterase activity, morphological changes in lysosomes, and insufficient regulation of stress proteins [189].

Amphibians constitute an ideal model for studying NP toxicity due to their natural metamorphosis, which involves major cardiovascular and respiratory changes, including the disappearance of gills and development of lungs under pituitary and thyroid control [145]. In studies using CuO NPs, after 48 h, an increase in apoptosis and a significant reduction in cell proliferation were observed [190].

**Figure 9:**
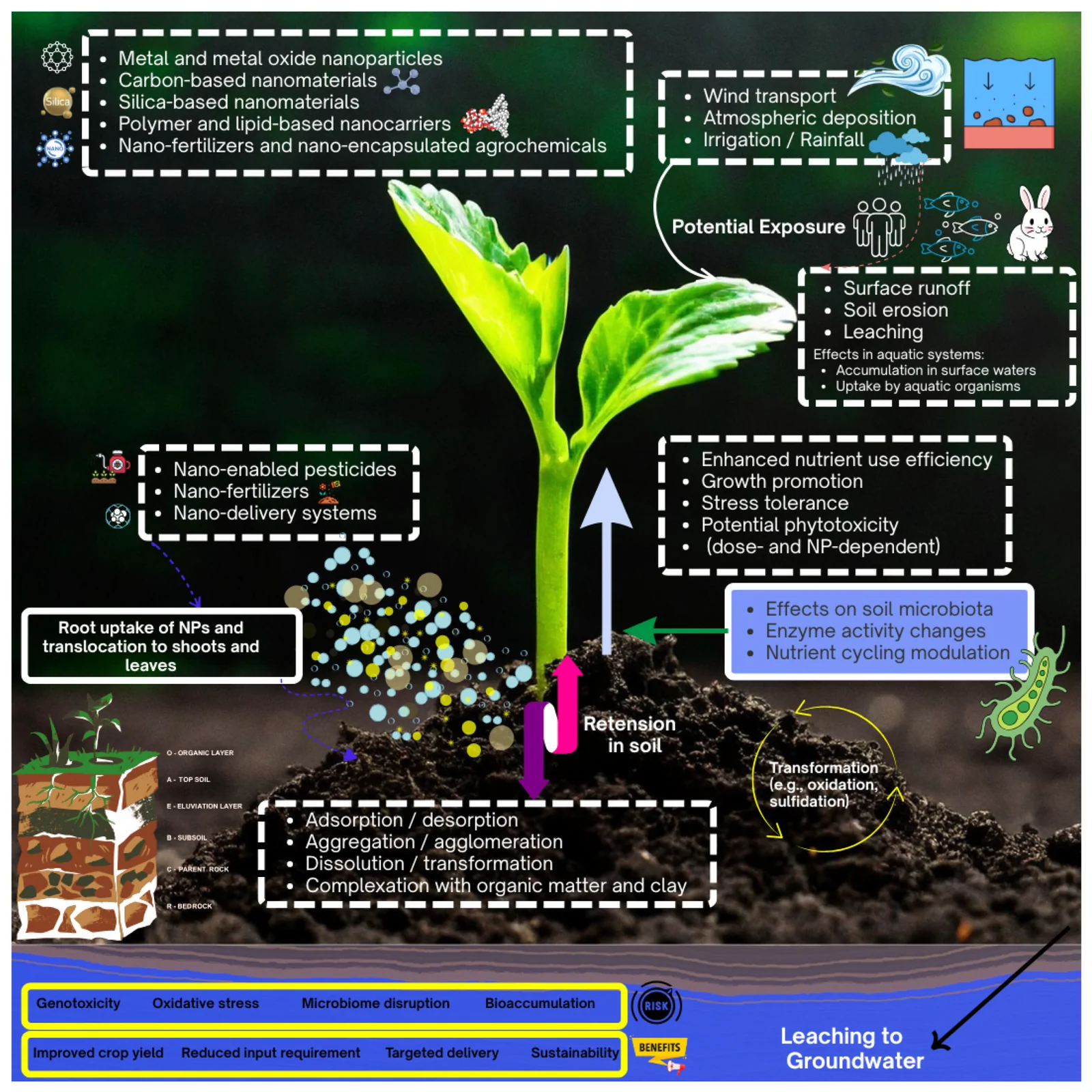
Schematic illustration of nanoparticle interactions in agricultural soil–plant–environment systems.

NPs are increasingly used in the production of everyday materials. The use of these materials causes NPs to be released into the environment, including soil, air, and water. Therefore, NPs represent a new source of risk that can have a serious impact on the lives of living organisms. With the current state of knowledge, the level of harmfulness of these particles cannot be clearly determined. The potential risk of using NPs is currently the subject of extensive research conducted by scientists from around the world. A schematic representation of nanoparticle transport and interactions within soil–plant–environment systems is shown in Figure 9.

This diagram illustrates the complex interactions and transport pathways of NPs within agricultural ecosystems, including soil, plants, water systems, and the surrounding environment, highlighting the interconnection between nanoparticle application, transformation, plant uptake, and dispersion into aquatic and atmospheric compartments, as well as the broader links between soil chemistry, plant physiology, and environmental transport processes.

### 5.4. Mechanisms of Nanoparticle Toxicity in Agricultural Systems

The increasing use of nanomaterials in agriculture has raised concerns regarding their potential toxicity to plants, microorganisms, animals, and humans. The toxicity of NPs depends on their physicochemical properties, including particle size, morphology, surface charge, chemical composition, concentration, solubility, aggregation behavior, and surface functionalization. Due to their small size and enhanced reactivity, NPs may exhibit greater biological effects than their bulk counterparts [191].

One of the primary mechanisms of NP toxicity is the generation of reactive oxygen species (ROS), leading to oxidative stress. Excessive ROS production may disrupt cellular redox balance, causing lipid peroxidation, protein denaturation, membrane damage, enzyme inactivation, and DNA damage [192,193]. Metal-based nanoparticles such as Ag, ZnO, CuO, and TiO_2_ are particularly associated with oxidative stress and cytotoxic effects.

Smaller particles generally show greater toxicity due to easier cellular penetration, while surface charge affects membrane interactions and intracellular transport. Aggregation and dissolution behavior additionally influence nanoparticle mobility, bioavailability, and environmental persistence [194]. Moreover, metal and metal oxide nanoparticles may release toxic ions, such as Ag^+^ and Zn^2+^, which can contribute to toxicity by interfering with enzymatic activity and other cellular processes [195].

In plants, nanoparticles may penetrate root tissues and translocate to aerial organs, potentially affecting seed germination, nutrient uptake, chlorophyll synthesis, photosynthesis, and overall plant growth. High NP concentrations may also induce mitochondrial dysfunction and genotoxic effects [196].

Nanomaterials can disturb soil ecosystems by negatively affecting microbial diversity, biomass, activity, and function. Their suppression of microbial biomass and soil enzyme activities may also hamper geochemical element cycling, potentially affecting soil environmental health and sustainable soil management [197].

In aquatic systems, nanoparticles originating from agricultural runoff may accumulate in water environments and affect algae, crustaceans, fish, and other organisms. Reported effects include oxidative stress, immune dysfunction, developmental abnormalities, and bioaccumulation within food chains [195].

From a human health perspective, chronic exposure through inhalation, ingestion, or dermal contact remains a concern. Some nanoparticles may cross biological barriers, including the blood–brain and placental barriers, potentially contributing to inflammation, neurotoxicity, and genotoxicity [164,165,166]. However, knowledge regarding long-term exposure under realistic agricultural conditions remains limited.

Overall, nanoparticle toxicity results from complex interactions between nanomaterial properties, environmental conditions, and biological systems. Understanding these mechanisms is essential for the safe and sustainable application of nanotechnology in agriculture.

### 5.5. Ecological Risk Assessment of Nanomaterials

Ecological risk assessment (ERA) of nanomaterials in agricultural systems is a complex, multi-stage process that integrates information on nanoparticle physicochemical properties, environmental fate, exposure pathways, and toxicological effects on different trophic levels. Unlike conventional chemicals, nanomaterials exhibit size-dependent behavior, surface reactivity, and transformation dynamics, which require adapted and nanospecific assessment frameworks [198].

The first step in ecological risk assessment involves hazard identification, which focuses on determining the intrinsic toxicity of nanomaterials toward representative environmental organisms, including soil microorganisms, plants, invertebrates, and aquatic species. Toxicological endpoints commonly include oxidative stress, membrane damage, genotoxicity, enzyme inhibition, and reproductive impairment. Metal-based NPs such as Ag induce oxidative stress via ROS generation, causing cellular dysfunction [199].

The second step is exposure assessment, which evaluates the environmental concentration and distribution of nanomaterials following their application in agriculture. Nanoparticles may enter the environment through fertilizer application, pesticide sprays, seed coatings, or irrigation systems. Their transport is influenced by soil properties (pH, organic matter content, texture), climatic conditions, and nanoparticle-specific properties such as aggregation tendency and dissolution rate. NPs can migrate through soil profiles, potentially affecting subsurface waters, while runoff and atmospheric deposition can contribute to their transport into agricultural soils [200].

The third component is fate and transformation analysis, which describes the physicochemical changes in nanomaterials in environmental compartments. Processes such as agglomeration, adsorption to clay minerals, redox reactions, and dissolution significantly affect nanoparticle bioavailability and toxicity. For example, AgNPs may release Ag^+^ ions, which are often more toxic than the nanoparticle form itself, while TiO_2_ nanoparticles can undergo surface modification under UV radiation, altering their reactivity [201].

The integration of hazard and exposure data enables risk characterization. Life-cycle-based frameworks, such as those coupling the USEtox model with nano-specific fate models (e.g., SimpleBox4Nano), derive characterization factors and impact scores to quantify ecotoxicological risk across a nanomaterial’s full life cycle [202]. However, uncertainty remains high due to limited field data and the predominance of short-term laboratory studies.

In addition to classical ERA approaches, recent studies emphasize the importance of multi-compartment and food-web-based risk assessment, considering the potential for nanoparticle bioaccumulation and trophic transfer. Metallic nanoparticles have been shown to transfer from primary producers to consumers along aquatic food chains, raising concerns about long-term ecological impacts and eventual human exposure [195].

To improve reliability, modern ecological risk assessment frameworks increasingly integrate life cycle assessment (LCA) and probabilistic modeling approaches, allowing for the evaluation of environmental impacts across production, application, and disposal stages. Moreover, coupling experimental toxicity data with computational modeling (e.g., QSAR and machine learning approaches) enhances prediction accuracy for poorly studied nanomaterials [203].

Overall, ecological risk assessment of nanomaterials in agriculture requires a holistic and interdisciplinary approach that combines environmental chemistry, ecotoxicology, and modeling tools. The development of standardized testing protocols and field-based validation studies is essential to reduce uncertainty and support the safe implementation of nanotechnology in agricultural systems.

### 5.6. Long-Term Effects on Food Chains and Human Health

The scale of nanomaterial release into terrestrial and aquatic environments over the long term remains difficult to quantify precisely, given persistent uncertainties in global emission estimates across the nanomaterial life cycle—from manufacturing through use and disposal [204]. A major concern is the bioaccumulation and biomagnification of nanoparticles along food chains. Metal-based nanoparticles such as Ag, ZnO, and TiO_2_ may accumulate in plant tissues and enter edible plant parts, raising concerns about dietary exposure in humans and livestock [205]. Once incorporated into biological systems, nanoparticles may interact with proteins, lipids, and nucleic acids, potentially disrupting cellular metabolism.

In the soil–plant system, nanoparticles may harm soil microorganisms and plants and subsequently affect consumers, including animals and humans. Some nanoparticles also accumulate in plant tissues, including edible parts, while the food-chain consequences of their bioaccumulation and long-term exposure remain poorly understood [148]. This pattern extends to aquatic environments, where agricultural runoff may further transport nanoparticles into planktonic organisms, reinforcing food-chain exposure discussed above [195].

Beyond the general exposure routes already discussed, experimental studies indicate that some nanoparticles may cross biological barriers and be transported to internal organs such as the liver and kidneys [171]. Prolonged exposure has been associated with oxidative stress, inflammation, and potential genotoxic effects, although toxicity depends strongly on nanoparticle properties and exposure conditions [206].

Despite increasing research, comprehensive data on chronic nanoparticle exposure remain limited. Most studies have focused on short-term cellular effects, with long-term biological consequences—such as disruption of lipid, glucose, and protein metabolism—only recently beginning to be explored [207]. In addition, nanoparticle transformations such as dissolution, aggregation, and protein corona formation may further influence their toxicity and environmental behavior [208].

Overall, further long-term and field-based studies are required to better understand the effects of nanomaterials on food chains, ecosystem stability, and human health.

### 5.7. Risk Management and Mitigation Strategies for Nanomaterials in Agriculture

The increasing application of nanomaterials in agriculture, including nano-enabled pesticides, fertilizers, and delivery systems, necessitates the development of effective risk management and mitigation strategies. While nanotechnology offers significant benefits in terms of crop productivity, nutrient efficiency, and pest control, its potential environmental and human health risks require a precautionary and systematic approach.

One of the key strategies for mitigating risks associated with nanomaterials is the implementation of regulatory frameworks and standardized testing protocols. International organizations such as the European Chemicals Agency (ECHA) and the Organisation for Economic Co-operation and Development (OECD) emphasize the need for nanospecific risk assessment methods, including evaluation of particle size distribution, surface chemistry, and environmental persistence [209,210]. The “safe-by-design” concept is increasingly promoted, aiming to engineer nanomaterials with reduced toxicity while maintaining their functional performance in agricultural applications.

To operationalize these principles, several international and national regulatory frameworks have been established to ensure the safe use of nanomaterials in agricultural systems.

At the international level, the OECD Working Party on Manufactured Nanomaterials (WPMN) provides harmonized guidance for testing methods, hazard identification, and risk assessment of engineered nanomaterials, including those applied in agrochemical formulations [211,212].

In the European Union, nanomaterials are regulated under the Registration, Evaluation, Authorisation and Restriction of Chemicals (REACH) framework, which was amended in 2018 to explicitly address nanoforms of substances. The European Chemicals Agency (ECHA) provides detailed guidance for the characterization and risk assessment of nanomaterials to support registrants in meeting these requirements [213]. Furthermore, Regulation (EU) 2019/1009 on EU fertilising products contains no nanomaterial-specific provisions; CE-marked fertilisers containing nanomaterials are therefore subject to the same general requirements as other EU fertilising products, including the obligation not to present a risk to human, animal or plant health, to safety or to the environment (Article 4(2)) [214].

In the United States, the Environmental Protection Agency (EPA) regulates nanoscale chemical substances under the Toxic Substances Control Act (TSCA), requiring premanufacture notification, information-gathering, and exposure assessment for new and existing nanomaterials, including those used in general agricultural and industrial products [215].

At the global level, the Food and Agriculture Organization (FAO) and the World Health Organization (WHO) have emphasized the importance of robust risk assessment, international collaboration, stakeholder engagement, and transparency in nano-enabled food and agricultural systems [216].

In addition, several countries have introduced national regulatory approaches. China’s National Technical Committee on Nanotechnology (SAC/TC279), under the Standardization Administration of China, has developed technical standards covering nanomaterial terminology, characterization, and safety since 2005 [217]. Similarly, in India, regulation of nanotechnology-based agricultural products falls under the joint purview of the Department of Biotechnology (DBT), which has issued draft guidelines for evaluation of nano-agri-input and food products, and the Ministry of Environment, Forest and Climate Change (MoEFCC), which regulates hazardous substances including nanomaterials under the Environment (Protection) Act, 1986 [218].

Overall, these frameworks demonstrate a growing global effort toward harmonizing nanomaterial regulation. However, specific guidelines for nano-enabled fertilizers and pesticides remain fragmented, highlighting the need for more agriculture-focused and internationally harmonized regulatory standards.

Beyond regulatory measures, environmental monitoring and life cycle assessment (LCA) are essential components of nanomaterial risk management. LCA enables the identification of potential release pathways during production, application, and disposal stages. However, measured data on nanomaterial concentrations and emissions in soil, water, and air remain scarce, and released nanomaterials may transform during their life cycle; improved environmental measurement data are therefore needed to assess their transformation, exposure, and long-term effects [219].

From an occupational health perspective, agricultural workers represent a high-risk group due to direct exposure to nanoparticle-containing formulations. Therefore, the use of personal protective equipment (PPE), including respirators, gloves, and protective clothing, is essential to minimize inhalation and dermal exposure. In addition, training programs and standardized handling protocols should be implemented to reduce accidental exposure [220,221].

Another promising mitigation strategy involves the development of nanoparticles synthesized using eco-friendly, biological methods. Green synthesis approaches using plants and microorganisms such as bacteria, fungi, and algae offer a simpler and more eco-friendly production route than other nanoparticle production processes [222]. Green-synthesized Ag NPs are typically capped with biocompatible, biodegradable biomolecules rather than synthetic coatings, which can reduce the release of toxic metal ions and promote faster environmental degradation, generally resulting in lower ecotoxicity compared to chemically synthesized nanoparticles [223]. In parallel, encapsulation technologies and controlled-release systems contribute to reduced application doses and minimized environmental contamination.

Finally, effective risk governance requires interdisciplinary collaboration between material scientists, toxicologists, environmental scientists, and regulatory authorities. Such cooperation is essential for establishing harmonized global guidelines and ensuring the sustainable development of nanotechnology-based agricultural solutions.

Overall, the implementation of comprehensive risk management strategies is crucial to balance the benefits of nanotechnology in agriculture with its potential risks to human health and ecosystems.

### 5.8. Standardized Risk Assessment Workflow for Nanoparticles in Agricultural Systems

To address the need for a harmonized approach to evaluate the safety of NPs in agricultural environments, a standardized risk assessment workflow is proposed. This framework integrates physicochemical characterization, exposure assessment, hazard identification, and environmental risk characterization in a structured stepwise manner.

The proposed workflow consists of the following stages:

1. Nanoparticle characterization: at the initial stage, nanoparticles should be comprehensively characterized in terms of size distribution, morphology, surface charge, chemical composition, aggregation behavior, solubility, and coating properties. These parameters are critical for predicting environmental behavior and biological interactions.

2. Exposure assessment: this step involves identifying potential release pathways of nanoparticles into agricultural systems, including soil application (fertilizers and pesticides), foliar sprays, irrigation water, atmospheric deposition, and accidental release during production or handling. Exposure concentration, frequency, and duration should be quantified under realistic field conditions.

3. Environmental fate and transport analysis: the transport, transformation, and persistence of nanoparticles in soil–water–plant systems are evaluated. Processes such as adsorption to soil particles, dissolution, aggregation, redox transformations, and interactions with organic matter determine their mobility and bioavailability.

4. Hazard identification and dose–response assessment: ecotoxicological and toxicological studies are used to assess potential adverse effects of nanoparticles on soil microorganisms, plants, aquatic organisms, and mammals. Dose–response relationships should be established for key endpoints such as oxidative stress, genotoxicity, growth inhibition, and reproductive toxicity.

5. Risk characterization: risk is determined by integrating exposure and hazard data to evaluate the likelihood and severity of adverse effects under specific environmental scenarios. This step enables classification of nanoparticles into low-, medium-, or high-risk categories for agricultural applications.

6. Risk management and decision-making: based on the risk characterization results, appropriate mitigation strategies are implemented, including safe-by-design nanomaterials, dose optimization, encapsulation techniques, regulatory restrictions, and monitoring programs.

Overall, this standardized workflow provides a structured framework for evaluating the environmental and health safety of nanoparticles in agriculture. It enables comparability between studies, supports regulatory decision-making, and facilitates the development of safer nano-enabled agricultural technologies.

### 5.9. Economic Analysis of Nanotechnology Products

**Figure 10:**
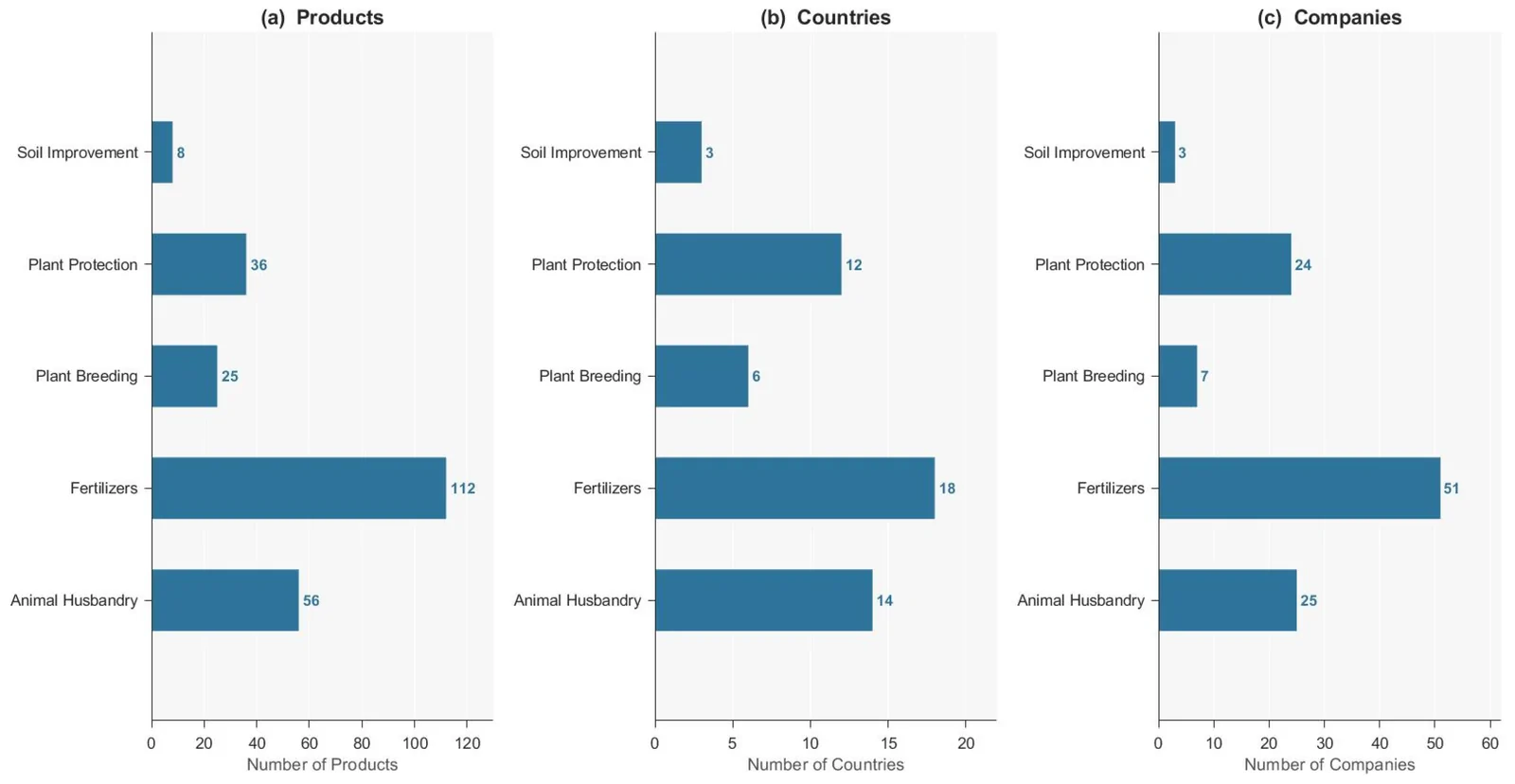
Distribution of nanotechnology applications in agriculture across different sectors: (**a**) Number of agricultural products utilizing nanotechnology, (**b**) involvement of countries in nanotechnology-driven agriculture, and (**c**) participation of companies in nanotechnology-based agricultural innovations. Based on: [224].

Figure 10a–c shows a comparative analysis of agricultural industrial products, countries, and companies associated with nanotechnology applications in agriculture. The agricultural industrial sector, as a vast network of interrelated activities, plays a crucial role in sustaining global food production. In recent years, the integration of nanotechnology has significantly enhanced the profitability and efficiency of agricultural processes, particularly through value-added innovations [224].

Figure 10a illustrates the distribution of agricultural products that incorporate nanotechnology, highlighting the dominance of fertilizers, which account for 112 cases. This significant number underscores the widespread application of nanotechnology in enhancing soil nutrients and improving crop yield efficiency. Animal husbandry follows with 56 cases, reflecting the growing adoption of nanotechnology in livestock nutrition, disease prevention, and overall farm productivity. Plant protection, with 36 cases, indicates the industry’s focus on using nanomaterials for pest and disease control, reducing chemical pesticide dependency. Meanwhile, plant breeding (25 cases) and soil improvement (8 cases) exhibit lower representation, suggesting that although nanotechnology plays a role in these areas, its commercialization and large-scale implementation are still developing [224].

Figure 10b presents the involvement of different countries in nanotechnology-driven agriculture, reinforcing the widespread global interest in these advancements. Fertilizers again lead, with 18 countries engaged in their nanotechnology-enhanced development, reflecting a shared priority in optimizing plant nutrition. Animal husbandry and plant protection follow, with 14 and 12 countries, respectively, participating in these applications. This distribution signifies an international effort to enhance livestock management and crop protection through nanotechnology. In contrast, plant breeding (6 countries) and soil improvement (3 countries) remain less prominent on a global scale, indicating that these fields may require further research and investment to achieve broader adoption [224].

Figure 10c depicts the number of companies actively investing in nanotechnology for agricultural products, aligning with the trends observed in Figure 10a,b. Fertilizers, with 51 companies, continue to dominate, highlighting the private sector’s recognition of nanotechnology’s potential in optimizing crop growth. Animal husbandry and plant protection, with 25 and 24 companies, respectively, demonstrate considerable industry involvement, suggesting strong market demand for innovations in these sectors. However, plant breeding (7 companies) and soil improvement (3 companies) attract significantly fewer investors, implying that technological and economic challenges may still hinder large-scale commercial adoption [224]. Overall, these data demonstrate the growing role of nanotechnology in agriculture, particularly in fertilizers, plant protection, and animal husbandry. The data suggest that while these areas receive substantial attention from both companies and countries, plant breeding and soil improvement remain underdeveloped. These insights underscore the need for continued research and investment to expand nanotechnology’s benefits across all agricultural sectors, ultimately contributing to more efficient and sustainable food production systems.

### 5.10. Commercial Nano-Enabled Fertilizer and Pesticide Formulations

In addition to research and industrial analyses, several nano-enabled agrochemical formulations are already commercially available and applied in modern agriculture. Nano-fertilizers are designed to improve nutrient use efficiency and reduce environmental losses compared with conventional fertilizers [107].

One example of a commercially available nano-fertilizer is the nano-urea formulation developed by the Indian Farmers Fertiliser Cooperative Limited (IFFCO). This product is designed to improve nitrogen use efficiency and reduce fertilizer runoff losses. Nano-urea is supplied as a liquid formulation, enabling efficient nitrogen uptake via foliar application and improving crop productivity while reducing environmental impact.

Another group of nano-enabled fertilizers includes nano-chelated formulations; for example, foliar application of nano-potassium chelate had a beneficial effect on basil production [112].

In the field of plant protection, nano-encapsulated pesticide formulations are used to improve the stability and controlled release of active ingredients [59]. Such formulations include nano-versions of insecticides, where active compounds are encapsulated in polymeric nanocarriers to increase their affinity to the target [22,59].

Nano-emulsions are also used as nanopesticide delivery systems, improving the dispersion and wettability of agricultural formulations [22].

Overall, the availability of commercial nano-enabled agrochemical products demonstrates the transition of nanotechnology from laboratory research to practical agricultural applications. However, despite their advantages, continuous monitoring of their environmental behavior and long-term ecological impacts remains necessary.

### 5.11. Artificial Intelligence and Nanotechnology in Agricultural Industries

With the advancement of AI technologies, new opportunities arise for optimizing the use of nanomaterials in agriculture. By harnessing data-driven insights, machine learning (ML) facilitates the precise deployment of nano-enabled solutions, improving crop yields, reducing input waste, and enhancing sustainable agricultural practices. ML algorithms process vast datasets from sensors and imaging technologies to detect plant diseases, predict yield outcomes, and recommend tailored interventions, thereby accelerating the adoption of nanotechnology in modern agriculture [225]. For example, ML-based decision-support systems are increasingly used to optimize fertilizer and nanoparticle application rates by predicting crop nutrient demand under variable soil and climate conditions, allowing site-specific dosing that minimizes environmental losses while maintaining optimal plant growth performance. In addition, predictive models are applied to estimate crop yield based on spectral imaging and soil sensor data, enabling farmers to adjust nano-fertilizer delivery strategies in real time.

**Figure 11:**
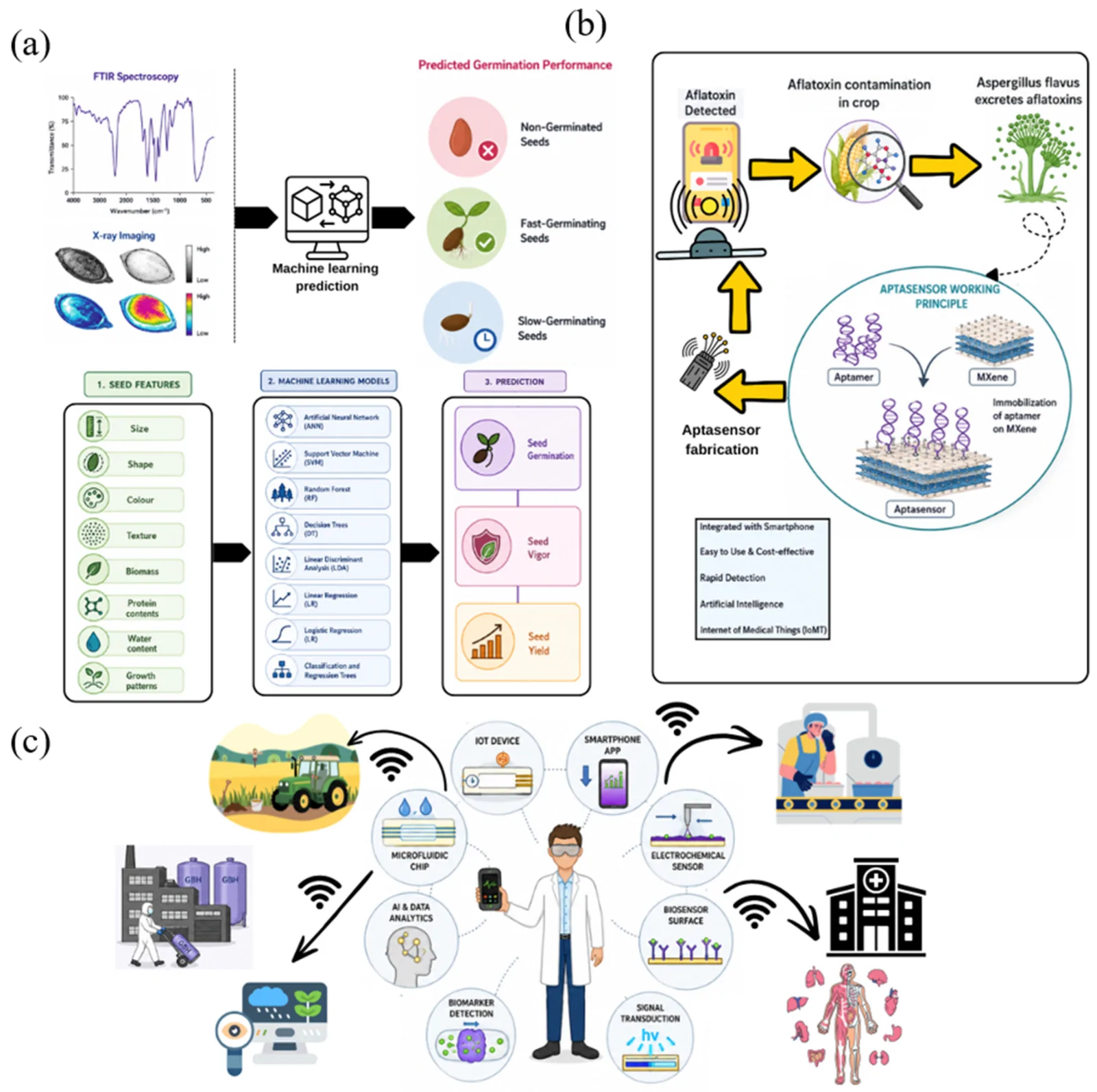
The conceptual outputs of ML applications for: (**a**) classification of nanoprimed seeds [226], (**b**) detecting aflatoxins [227], and (**c**) GLP detection [228].

The study by Shelar et al. [226] discusses the application of machine learning in the agricultural industry, specifically for the detection and classification of nanoprimed seeds to improve crop production. According to Figure 11a, the machine learning technology is used for post-seed nanopriming diagnosis to predict nanoprimed seed viability and analyze phenotypic characteristics. The study highlights how artificial intelligence and machine learning, combined with advanced computer vision techniques, can automate seed diagnostics and enhance the accuracy of seed germination assessments.

According to Figure 11b, Parihar et al. applied ML in the agricultural sector through the development of MXene-based Aptasensors for detecting aflatoxins in food and agricultural products. Their work integrated computational approaches, including artificial intelligence and machine learning, to enhance biosensing technologies. The study emphasized the role of ML in advanced data analysis, optimizing sensor design, and enabling IoT-integrated real-time detection devices [227], while smart sensor systems for detecting glyphosate in agriculture have also been investigated in the context of precision farming using machine learning. Mazuryk et al. [228] highlighted the environmental risks associated with excessive glyphosate use and the need for real-time monitoring tools. ML-based algorithms enhance the accuracy of glyphosate detection by processing large datasets, enabling precise site-specific herbicide application. The integration of ML with smart materials and nanosensors allows for advanced prediction models, improving agricultural sustainability. These technologies contribute to reducing environmental contamination while optimizing crop management through efficient pesticide usage and enhanced decision-making capabilities [228].

### 5.12. Research Limitations and Future Directions

Although many studies on nanomaterials in agriculture are currently conducted under controlled short-term laboratory conditions due to their reproducibility and cost efficiency, this trend is likely to continue in the near future. However, there is a growing recognition that such approaches are insufficient for fully understanding the environmental fate and ecological impacts of nanomaterials.

Field-scale, long-term, and multi-season studies are therefore essential to capture the complexity of real agricultural systems. Evidence from selected field trials indicates that repeated application of nanoparticle-based fertilizers may lead to gradual accumulation in soils, influencing microbial diversity, enzymatic activity, and nutrient cycling processes.

In addition, mesocosm and long-term experiments demonstrate that nanoparticle mobility and bioavailability are highly dependent on environmental factors such as soil heterogeneity, organic matter content, irrigation practices, and seasonal variability. These findings highlight a critical limitation of laboratory-based toxicity assessments, which often fail to reproduce realistic environmental conditions.

Consequently, integrated research approaches combining laboratory, mesocosm, and field-scale experiments are necessary to improve the reliability of environmental risk assessment of nanomaterials in agricultural systems.

To facilitate clarity and comparability of nanomaterial effects across different biological systems, a summary table is provided below. Table 4 includes the type of nanoparticles, tested organisms, exposure concentrations, and the observed biological or environmental effects. This structured overview allows for a direct comparison of toxicity outcomes reported in different studies and highlights the variability of nanoparticle responses depending on biological system and exposure conditions.

## 6. Future Perspectives

Despite the rapid development of nanotechnology in agriculture, several scientific, technological, environmental, and regulatory challenges remain insufficiently addressed. Although numerous studies have demonstrated the potential benefits of nano-enabled fertilizers, pesticides, and delivery systems, long-term field-scale data remain scarce. Consequently, the environmental fate, persistence, and bioaccumulation of NPs in realistic agricultural ecosystems remain poorly understood.

Future research should therefore prioritize long-term, multi-season, and field-scale investigations to better evaluate the ecological and toxicological impacts of nanomaterials under realistic environmental conditions. Particular attention should be given to the interactions between NPs and soil microbiota, nutrient cycling processes, groundwater systems, and trophic transfer within food chains. In addition, standardized experimental methodologies and harmonized toxicity assessment protocols are required to improve comparability between studies and support regulatory decision-making.

Another important research direction involves the development of biodegradable, green-synthesized, and “safe-by-design” nanomaterials with reduced environmental persistence and lower ecotoxicity. The optimization of nanoparticle surface properties, release kinetics, and targeted delivery mechanisms may significantly improve agricultural efficiency while minimizing unintended environmental effects.

The integration of artificial intelligence (AI), machine learning (ML), and precision agriculture technologies is also expected to play an increasingly important role in future nano-agricultural systems. AI-assisted predictive models may support optimization of nanoparticle dosage, nutrient delivery scheduling, crop yield estimation, and environmental monitoring, thereby improving sustainability and reducing excessive agrochemical application.

From a regulatory perspective, harmonized governance frameworks specifically dedicated to nano-enabled agricultural products are still lacking, highlighting the need for greater international collaboration between scientists, regulatory agencies, and policymakers.

Overall, future advancements in agricultural nanotechnology should aim to balance technological innovation with environmental safety, economic feasibility, and public acceptance in order to support sustainable global food production systems.

## 7. Conclusions

Nanotechnology is one of the fastest-developing fields of science, creating unlimited opportunities for many spheres of human life. Its many applications are proof of this. It is through nanotechnology that various structures of nanomaterials are obtained whose physical, chemical and biological properties differ from those of the same materials found on a macro scale. However, these unique properties also introduce new and still insufficiently understood risks related to their environmental persistence, bio-reactivity, and transport across ecological compartments.

Solutions allowing the use of NPs in agriculture and the food industry have appeared relatively recently. The main advantages of nanotechnology include increased plant efficiency and resistance to abiotic stress. However, despite these benefits, comprehensive long-term studies addressing environmental fate, chronic exposure, and human health impacts of nanomaterials are still limited. Therefore, further scientific research should focus on generating comprehensive knowledge about the toxicity of specific NPs on the environment to implement them in sustainable agriculture. Many studies have demonstrated the harmful effects of NPs on animals, as foreign physicochemical properties introduced into living organisms can disturb natural physiological mechanisms, impair reproduction, and hinder development. Nanoparticle exposure has also been associated with morphological abnormalities, altered cellular function, and neuronal damage. Due to the increasing accumulation of NPs in inland waters and oceans, research is expanding to study their impacts on aquatic organisms, particularly fish, Daphnia, sea urchins, plankton, crustaceans, and mollusks. These findings highlight that nanoparticle effects are highly species-specific and strongly dependent on dose, exposure route, and environmental conditions, which complicates risk extrapolation across ecosystems.

The interactions between nanomaterials and living organisms are complex. Evaluating human exposure to nanomaterials during their production and use has become a significant challenge. It is also a problem to understand their physicochemical properties, including concentration, particle size, and specific surface area, as these properties depend on many external factors such as the characteristics of living organisms (e.g., species, pH, and life cycle phase). There is a need to study nanomaterials in different environments, i.e., air, water and soil, and during their life cycle (production, processing, use, and disposal). In particular, standardized field-based studies and harmonized testing protocols are still lacking, which limits comparability between existing datasets. After conducting a literature study, it can be affirmed that the use of nanomaterial-based agrochemicals by farmers is steadily increasing, although large-scale adoption remains limited and is still transitioning from research to widespread commercial application.

Traditional agrochemicals may gradually be replaced by nano-based agents. More detailed research on trophic-level and particle-level contaminant dynamics needs to be conducted. Conducting studies in order to identify research gaps is an urgent global need. Despite two decades of research, field-scale and long-term studies remain insufficient. One of the goals for future research should be to study nanomaterial behavior under realistic environmental conditions, supporting the transition of these technologies toward commercial-scale application. Bridging the gap between laboratory-scale experiments and real-world agricultural conditions is therefore a key priority for future research. Despite their many benefits, nanomaterials remain incompletely understood and should therefore be treated as potential risk factors, particularly for human health and the environment. In addition to promoting nanotechnology, it also seems reasonable to conduct public consultations on its future and its impact on the environment. Finally, the development of robust regulatory frameworks and risk assessment methodologies is essential to ensure that the deployment of nanotechnology in agriculture remains both effective and environmentally safe.

## Figures and Tables

**Table 1:** Classification and characteristics of selected nanomaterials used in agriculture.

Nanomaterial Type	Structural Characteristics	Key Physicochemical Properties	Typical Agricultural Applications
Carbon nanotubes (CNTs)	Cylindrical graphene-based nanostructures; single-walled (SWCNTs) or multi-walled (MWCNTs)	High mechanical strength, electrical conductivity, large surface area	Nanosensors, pesticide delivery, plant growth stimulation
Quantum dots (QDs)	Semiconductor nanocrystals	Unique optical and fluorescent properties, high photostability	Biosensing, pathogen detection, imaging applications
Nanorods	Rod-shaped nanoparticles with high aspect ratio	Enhanced optical and catalytic properties	Targeted agrochemical delivery, biosensors
Core-shell nanoparticles	Nanoparticles composed of an inner core surrounded by an outer shell layer	Tunable surface chemistry, improved stability, controlled release	Controlled-release fertilizers and pesticides
Nanoemulsions	Colloidal dispersions of nanosized oil/water droplets	High dispersion stability, enhanced solubility and bioavailability	Nanopesticides, herbicide formulations
Nanogels	Three-dimensional hydrogel nanoparticles	High water retention, stimuli-responsive release	Controlled nutrient delivery, moisture retention
Metal nanoparticles (Ag, Cu, Au)	Metallic nanoparticles	Antimicrobial activity, catalytic properties	Antibacterial agents, fungicides, crop protection
Metal oxide nanoparticles (ZnO, TiO_2_, Fe_3_O_4_)	Oxide-based nanostructures	Photocatalytic activity, ROS generation	Nano-fertilizers, pathogen control, soil remediation, nutrient supply
Polymeric nanoparticles	Biodegradable polymer-based nanocarriers	Controlled release, low toxicity, high encapsulation efficiency	Agrochemical delivery systems
Lipid-based nanoparticles	Lipid vesicles or lipid-core nanostructures	Biocompatibility, improved penetration and stability	Delivery of pesticides, herbicides, and nutrients

**Table 2:** The list of selected NPs for pesticide production.

Nanomaterials	Active Ingredients	Benefits	References
MA-chitosan nanogel	*Cuminum cyminum*	Effective against: *Sitophilus granarius* and *Tribolium confusum*	[80]
Alginate–chitosan nanocapsules	Acetamiprid	Superior in controlled release compared to commercial formulation	[81]
(PEGs)-based functionalized amphiphilic copolymers nanomicelles	Carbendazim	Effective against: *Rhizoctonia solani*, Enhanced controlled release	[82]
Pluronic F-127 micelle-forming block copolymers	Geranylphenols	Effective against: *Botrytis cinerea* Increased pesticide activity	[83]
Solid Lipid NPs	Atrazine, simazine	Effective against: *Raphanus raphanistrum* High encapsulation efficiencies, Good physicochemical stability Safe for plant growth	[84]
Porous silica NPs	Abamectin	Enhanced controlled release Increasing photostability Enhanced solubility	[85]
Inclusion complexes (BC/SHECD−LDH)	Beta-cypermethrin	Enhanced controlled release	[86]
MWCNT-g-PCA	Zineb, Mancozeb	Effective against: *Alternaria alternata* Enhanced solubility	[87]
CM-β-CD-MNPs-Diuron	Diuron	Slow release	[88]
Nanoencapsulated Eucalyptus extracts	*Eucalyptus globulus*	Effective against: *Myzus persicae*	[89]
ZnO and ZnTiO_3_ embedded in organic polymers	-	Antifungal against *Aspergillus niger* in a non-agricultural (stone conservation) setting	[90]
Chitosan-magnesium nanocomposite	Chitosan, Magnesium	Effective against: *Acidovorax oryzae* and *Rhizoctonia solani*	[91]
Nanostructured alumina	-	Effective against: *Sitophilus oryzae*	[92]
MeSiNPs@bPEI	Citridiol	Sustainable release of biopesticides	[93]
Polymeric NPs	*Azadirachta indica*	Improved short-term phagodeterrence against *Spodoptera frugiperda*; limited residual effect	[94]
NGNF	*Azadirachta indica*	Effective against: *Helicoverpa armigera* and *Spodoptera litura*	[95]

**Table 3:** Toxicological effects of nanomaterials used in agriculture on animals, plants and human cells exposed to NPs.

NPs	Size (nm)	Exposure Route	Concentration	Effects	Target	References
ZnO	~38	Oral	300 and 2000 mg/kg	Histopathological lesions in liver and kidney tissues	Wistar rats	[170]
ZnO	~41	Oral	5000 mg/kg	Decreased body weight, pancreas, brain and lung weight increased	CD-1 (ICR) Mice	[171]
ZnO	<100	i.p. injection *(in vivo)*	PBMCs: 20, 40, 80 and 100 μg/mL Mice: 25, 50 and 100 mg/kg	Genotoxicity, oxidative stress, and apoptosis in PBMCs; clastogenicity, G0/G1 arrest, and DNA damage in bone marrow and liver	Human PBMCs (in vitro) and Swiss albino mice	[172]
ZnO	<50	Sediment	600 and 2000 mg/kg	Oxidative stress	*Caenorhabditis elegans*	[173]
ZnO	<100		10, 30, 60, 90, or 120 mg/L	Acute toxicity, Developmental abnormalities oxidative DNA damage	*Zebrafish embryos*	[174]
Cu		Plant agar media	200 to 1000 mg/L	Cu cross the cell membrane and agglomerate in the cells	*Phaseolus radiatus* *Triticum aestivum*	[175]
CuO	<50	Solid matrix	500 mg/kg	Reduced root growth, Reduced shoot growth	*Triticum aestivum*	[176]
TiO_2_	20	Soil	0 to 100 mg/kg	Up to 60 mg/kg favoured plant growth, Beyond 60 mg/kg chlorophyll content was reduced and micronuclei formation	*Triticum aestivum*	[177]
Ag	~13	Water solution	1 and 10 mg/L	Toxicity is driven primarily by released Ag ions, Altered expression of several proteins	*Triticum aestivum*	[178]
ZnO	-	Water solution	10, 100, 500, and 1000 mg/L	Reduced length and number of roots	*Oryza sativa*	[179]
Ag	-	Whatman paper bridges in test tubes	0, 0.2, 0.5 and 1 mg/L	Reduction in root elongation, Excessive reactive oxygen species generation and cell death	*Oryza sativa*	[180]
ZnO	-	Water solution	1000 mg/L	Reduced root length	*Zea mays*	[181]
ZnO	<50	Filter paper in glass Petri dishes	100, 500 and 1000 ppm	Negative impact on both root and shoot length	*Cicer arietinum*	[182]
CuO	~7	Filter paper in glass Petri dishes	0.2–300 µg/mL	Decreased roots growth	*Lactuca sativa*	[183]
Al_2_O_3_	<50	Sand	1.25–5 µM	Chromosome aberrations	*Allium cepa*	[184]

**Table 4:** Effects of engineered nanomaterials in biological and environmental systems.

Nanoparticle Type	Organism/System	Exposure Concentration	Observed Effects	References
ZnO NPs	Rice (*Oryza sativa*)	25–100 mg/L	Growth inhibition, reduced biomass and chlorophyll content, oxidative damage (altered CAT, APX, SOD gene expression)	[229]
TiO_2_ NPs	Wheat (*Triticum aestivum*)	5-150 mg/L	Organ-dependent antioxidant responses; toxicity above 5 mg/L [230]; impaired photosynthesis (decreased chlorophyll a, PSII efficiency and net photosynthetic rate) [231]	[230,231]
Potassium nano-silica (PNS)	Maize (*Zea mays*), under drought stress	100–200 mg/L	Improved nutrient uptake (N, K, Cu, Mn, Si) and alleviated drought-induced declines in shoot/seed mineral content	[232]
Ag NPs	Soil bacteria	10–100 mg/kg	Microbiome disruption; AgNPs < Ag^+^ toxicity at low/mid doses	[233]
CuO NPs	Human hepatocellular carcinoma cells (HepG2)	2–50 µg/mL	Cytotoxicity, apoptosis, mitochondrial damage	[234]
Fe_3_O_4_ NPs	Maize (*Zea mays* soil system)	50–500 mg/kg	Increased soil dehydrogenase enzyme activity (+84.9% at 500 mg/kg); increased root length, no effect on biomass/photosynthesis	[235]
MWCNTs (pristine and COOH-functionalised)	Human lung cells	1–40 µg/mL	Inflammation, oxidative DNA damage (Fpg-comet), reduced cell viability	[236]
Nano-fertilizers (N, P-based)	Agricultural soil systems	Field application	Increased nutrient efficiency; reduced leaching	[237]

## Data Availability

No new data were created or analyzed in this study. This article is based on a comprehensive review of previously published literature. All data supporting the findings of this study are available within the article and in the cited references.
